# A comparison of the associations between adiposity and lipids in Malawi and the United Kingdom

**DOI:** 10.1186/s12916-020-01648-0

**Published:** 2020-07-16

**Authors:** Ana Luiza G. Soares, Louis Banda, Alemayehu Amberbir, Shabbar Jaffar, Crispin Musicha, Alison J. Price, Amelia C. Crampin, Moffat J. Nyirenda, Deborah A. Lawlor

**Affiliations:** 1 MRC Integrated Epidemiology Unit, University of Bristol, Bristol, UK; 2 Population Health Sciences, Bristol Medical School, University of Bristol, Oakfield House, Oakfield Grove, Bristol, BS8 2BN UK; 3 Malawi Epidemiology and Intervention Research Unit (MEIRU), Lilongwe and Malawi, Malawi; 4 Partners in Hope, Lilongwe, Malawi; 5 Department of Medicine, University of California Los Angeles David Geffen School of Medicine, University of California Los Angeles, Los Angeles, USA; 6 Department of International Public Health, Liverpool School of Tropical Medicine, Liverpool, UK; 7 Faculty of Epidemiology and Public Health, London School of Hygiene & Tropical Medicine, London, UK; 8 Institute of Health and Wellbeing, University of Glasgow, Glasgow, UK

**Keywords:** Obesity, Body mass index, Waist-hip ratio, Lipid profile, Dyslipidaemia, Sub-Saharan Africa, ALSPAC

## Abstract

**Background:**

The prevalence of excess adiposity, as measured by elevated body mass index (BMI) and waist-hip ratio (WHR), is increasing in sub-Saharan African (SSA) populations. This could add a considerable burden of cardiovascular and metabolic diseases for which these populations are currently ill-prepared. Evidence from white, European origin populations shows that higher adiposity leads to an adverse lipid profile; whether these associations are similar in all SSA populations requires further exploration. This study compared the association of BMI and WHR with lipid profile in urban Malawi with a contemporary cohort with contrasting socioeconomic, demographic, and ethnic characteristics in the United Kingdom (UK).

**Methods:**

We used data from 1248 adolescents (mean 18.7 years) and 2277 Malawian adults (mean 49.8 years), all urban-dwelling, and from 3201 adolescents (mean 17.8 years) and 6323 adults (mean 49.7 years) resident in the UK. Adiposity measures and fasting lipids were assessed in both settings, and the associations of BMI and WHR with total cholesterol (TC), low-density lipoprotein cholesterol (LDL-C), high-density lipoprotein cholesterol (HDL-C) and triglycerides (TG) were assessed by sex and age groups in both studies.

**Results:**

Malawian female adults were more adipose and had more adverse lipid profiles than their UK counterparts. In contrast, Malawian adolescent and adult males were leaner and had more favourable lipid profiles than in the UK. Higher BMI and WHR were associated with increased TC, LDL-C and TG and reduced HDL-C in both settings. The magnitude of the associations of BMI and WHR with lipids was mostly similar or slightly weaker in the Malawian compared with the UK cohort in both adolescents and adults. One exception was the stronger association between increasing adiposity and elevated TC and LDL-C in Malawian compared to UK men.

**Conclusions:**

Malawian adult women have greater adiposity and more adverse lipid profiles compared with their UK counterparts. Similar associations of adiposity with adverse lipid profiles were observed for Malawian and UK adults in most age and sex groups studied. Sustained efforts are urgently needed to address the excess adiposity and adverse lipid profiles in Malawi to mitigate a future epidemic of cardio-metabolic disease among the poorest populations.

## Background

Excess adiposity, including high body mass index (BMI), waist circumference (WC) and waist-hip ratio (WHR), is no longer solely a problem of high-income countries (HIC) and is rising rapidly in low- and middle-income countries (LMIC), including in sub-Saharan Africa (SSA), with most SSA countries experiencing the double burden of undernutrition and obesity [1–3]. Evidence from HIC, from populations of white European origin, shows that higher BMI and measures of central adiposity, such as WHR and WC, are associated with adverse lipid profiles, including higher total cholesterol (TC), low-density lipoprotein cholesterol (LDL-C) and triglycerides (TG), which are key modifiable risk factors for coronary heart disease (CHD) [4–6]. The body of evidence on this relationship in SSA countries is growing and also showing associations between higher generalised and central adiposity with adverse lipid profile in both men and women [7–11] and adults from urban and rural area [9]. However, very few studies have made direct comparisons between SSA and HIC populations, which have a much more advanced stage of economic development. If greater adiposity is as strongly related to adverse lipid profiles in SSA populations as it is in HIC, the emerging obesity epidemic is likely to result in a considerable burden of CHD that these countries may be not equipped to prevent or treat.

Evidence suggests that African origin males have similar adiposity distributions to Europeans, whilst females are more adipose [12–15], yet people of African ancestry have also been shown to have a more favourable lipid profile [15–18]. Studies carried out in South Africa have shown that obesity was associated with TC, LDL-C and high-density lipoprotein cholesterol (HDL-C) in white, but not black women, and that associations of fat mass index and visceral adipose tissue (VAT) with lipids were stronger in white than black women, highlighting ethnic differences [7, 18]. However, little is known about how the association between adiposity and lipids in SSA compares with populations of white, European origin in HIC. Comparing methods, data and results between LMIC and HIC are important in gaining better understanding of how to prevent non-communicable diseases (NCD) in both LMIC and HIC, given there is often a wide variation in exposure levels and marked differences in underlying confounders of the associations between the risk factors and NCD [19, 20]. To our knowledge, few studies have taken this approach.

More than 20 years ago, a study comparing Cameroonian and French men and women found that age-adjusted associations of WC and WHR with fasting TC and TG (other lipids were not assessed) were stronger in French than in urban and rural Cameroon residents [14]. Since then, SSA countries have experienced a rapid epidemiological transition. BMI has steadily increased in Africa since 1980, especially in the northern and southern regions, with accelerated trends observed in women [21]. The increase in BMI in European and other HIC has been accompanied by a change in its distribution, with a right skew, such that even small incremental increases in mean BMI were associated with increased numbers with high BMI. If the same thing is happening and continues to happen in SSA as it experiences the ‘obesity epidemic’, then the future risk of cardiovascular diseases and poor health may be more marked. This is because a continuing right shift in the BMI distribution in these countries would potentially result in further increases in overweight and obesity without any notable reduction in underweight. Increase in life expectancy due to reductions in child mortality [22] and adult human immunodeficiency virus (HIV)-related mortality [23], as well as increase in sedentary behaviour, alcohol consumption, unhealthy diet and urbanisation [24–28], might have influenced the manifestation of adiposity and lipid profile in SSA populations and lead to differences in the association between adiposity and lipids, which could also influence how these differ from European populations.

In this study, we compare the distribution and strength of associations of general (BMI) and central (WHR) adiposity with lipid profiles in an urban Malawian population and a predominately urban UK population. There have been a number of reviews comparing WC and WHR associations with cardiovascular disease but their conclusions have varied in terms of differences in associations between WC and WHR and BMI, with the largest studies (with sufficient sample size to explore non-linear as well as linear associations) finding similar magnitudes of association for these adiposity indicators [29]. This was unsurprising given the strong correlations between some of these measurements. In a previous study of the Malawian cohort used here, we showed that the correlation between WC and BMI was very strong (0.83) whereas that between WHR and BMI was weak (0.15) [30]; therefore, WHR provides a more distinct measure from BMI, as a measure of body fat distribution, than would WC (which given the strong correlation with BMI would likely produce very similar associations and therefore add little to the manuscript).

## Methods

### Study design, setting and participants

The Malawi Epidemiology and Intervention Research Unit (MEIRU) conducted a population-based cross-sectional study in Southern Karonga district (rural setting) and Malawi’s central capital city, Lilongwe (urban setting), between May 2013 and April 2017. All adults aged 18 years and older, who were usually resident at a household in either study area and were able to consent, were eligible and invited to be included in the study (*n* = 33,177). Individuals who identified themselves as visitors were excluded. Details on recruitment and data collection have been previously described [9, 30–32]. Questionnaires were applied and anthropometric and blood pressure measures as well as blood samples were collected at the participant’s household by interviewers and nurses. Questionnaires were available in English, Chichewa (the main language of the Central Region) and Chitumbuka (the main language of the Northern Region), and the data were collected using Open Data Kit on Android operating system tablets. For this study, we only included urban participants who had complete data on anthropometry and fasting serum lipids, as the UK cohort to be compared was of residents living in an urban or sub-urban area. In order to have comparable age groups to the UK cohort, we restricted analyses to Malawians aged 18 to 19 (*n* = 1248, median age 19; interquartile range (IQR) 18 to 19) in both males and females, females aged 40 to 60 (*n* = 1487, median age 47; IQR 43 to 52) and males aged 43 to 70 (*n* = 790, median age 52; IQR 47 to 60). Data on the full population has been already published [9]. The underlying dataset without lipids is available through the London School of Hygiene and Tropical Medicine Research Data Compass <https://datacompass.lshtm.ac.uk/961/>.

Data from the UK Avon Longitudinal Study of Parents and Children (ALSPAC) was used as a comparator high-income, European population. Pregnant women resident in the Avon area of the UK with an expected delivery date between 1 April 1991 and 31 December 1992 were recruited [33, 34]. Since then, mothers, partners and children have been followed up regularly through questionnaires and clinical assessments [33, 34]. In this study, children who attended a follow-up when they were aged 18 years and mothers and fathers who attended assessments at around the same time and who had complete data on serum lipids and anthropometry were included. The assessments occurred between 2008 and 2011. Our analyses in ALSPAC included 3201 offspring (median age 17.8; IQR 17.6 to 17.9), 4423 mothers (median age 48; IQR 45 to 51) and 1900 fathers (median age 53; IRQ 50 to 56). The ALSPAC website contains details of all the data that are available through a fully searchable data dictionary and variable search tool <http://www.bristol.ac.uk/alspac/researchers/our-data/>.

### Data collection and definitions

In Malawi, a morning venepuncture sample, after a minimum 8-h fast, was collected to assess serum lipids (TC, LDL-C, HDL-C and TG). Participants who did not fast were re-visited once. The samples were transported and processed on the same day at the on-site project laboratory. Fasting lipids were assayed using the enzymatic method (Beckman Coulter Chemistry Analyser, model AU480). The two laboratories (one each in the rural and urban centres) participated in external (Thistle RSA system) and internal quality controls (exchanging samples between sites for repeat testing) [32]. Anthropometric measures were taken twice using standard protocols, and the mean of the two was used. Weight was measured using electronic scales (accuracy of 100 g), height was measured using portable stadiometers (accuracy of 1 mm) and waist and hip measurements were taken using a non-stretch metallic tape with a narrow blade and a blank lead-in. Participants were asked to remove shoes, outer clothing and headdresses. WC was measured on bare skin in the narrowest part of the abdomen between the ribs and iliac crest, and hip circumference was measured over light clothing at the widest part of the buttock.

In the UK, anthropometric measures were assessed twice at the clinic visit, and the mean of the two measures was used. Weight was measured using electronic scales to the nearest 100 g, and height was measured using a stadiometer and recorded at the nearest 1 mm. WC was measured at the mid-point between the lower rib and the iliac crest to the nearest 1 mm with a flexible tape measure. Hip circumference was measured of the trochanter major to the nearest 1 mm. Measures on waist and hip circumferences were only available for adults. This was because a large proportion of the offspring refused to have these measurements taken in a previous clinic (mean age 15) and so we did not collect it at the clinic used here. Blood samples were taken following a standardised protocol. Participants were instructed to fast overnight or for at least 6 h prior to their clinic visit. After collection, the blood was immediately centrifuged and frozen at − 80 °C. The samples were assayed 3–9 months later, with no previous freeze-thawing cycles. Plasma lipids were measured using the Lipid Research Clinics Protocol with enzymatic method (Technicon RA500), and the Friedewald equation [35] was used to calculate LDL-C concentration: LDL = TC − HDL - (TG/2.2).

Overweight was considered as BMI 25–29.9 kg/m^2^ and obesity as BMI ≥ 30 kg/m^2^ [36]. Central obesity was defined as WHR > 0.85 in females and WHR > 0.90 in males [36]. TC, LDL-C and TG were considered high if they were ≥ 5.2 mmol/L, 3.4 mmol/L and 1.7 mmol/L, respectively, and HDL-C was considered low if it was < 1.0 mmol/L [37], and any dyslipidaemia was considered as any of these conditions.

### Assessment of potential confounders in both cohorts

In Malawi, ethnicity, household assets score, education, marital status, parity (females), smoking status, alcohol intake, physical activity, lipid-lowering medication and HIV status were used as confounders. In the UK, ethnicity, family income, education (maternal education for adolescents), marital status (adults only), parity (female adults), smoking status, alcohol intake and lipid-lowering medication (adults only) were explored as potential confounders. Full details of the methods used to measure and categorise these variables are provided in Additional file 1: Supplementary methods.

### Statistical analysis

We internally age-standardised the anthropometric and lipid measures to enable comparisons between Malawi and the UK and described such measures in both settings, stratified by sex and age groups. We examined associations between continuous and binary measures of adiposity and lipids. Nonetheless, our main analyses use continuous measures, to increase precision of our estimates, and given ideal thresholds for defining obesity or dyslipidaemia may differ in the two populations, with lower cut-off points suggested for SSA men and similar or higher threshold for SSA women than those recommended by the World Health Organisation [38, 39]. We investigated the extent to which there was deviation from linear associations by assessing mean lipid levels by fifths of BMI and WHR measures in standard deviation (SD) units by sex and age group. We also used likelihood ratio tests to compare models using quintile categories of BMI and WHR to models using continuous BMI and WHR variables.

We used multivariable linear regression to examine the association of both BMI and WHR with each lipid measure, adjusted initially for age and then for the confounders (ethnicity, household assets score/family income, education, marital status, parity (females), smoking status, alcohol intake, physical activity (Malawi only), lipid-lowering medication and HIV status (Malawi only), with the units or categories for each of these as defined in Additional file 1: Supplementary methods). We present the results as mean difference in age-adjusted lipid level for a 1-SD (*z*-score) increase in BMI or WHR.

We compared age-standardised anthropometric and lipid measures, and differences between adjusted associations of anthropometric measurements with lipids, between Malawi urban and UK resident women and men by examining the point estimates and their 95% confidence intervals. Statistical evidence for sex differences within each age group and within each country were obtained from interaction tests.

Since the primary sampling unit in Malawi was the household and all individuals aged 18 years and older were eligible to be included in the study, we accounted for clustering by using robust standard errors in all regression models. The number of individuals living in the same household in each age/sex group is presented in Additional file 1: Table S1. For most groups, the vast majority of participants were the only included person from that household, with the largest proportion with two or more participants in the same household being adult males (24%).

The analyses were carried out in the software Stata 15.1® (Statcorp, College Station, TX, USA).

### Missing data

In Malawi, the only variable with missing data was HIV status (11.2% in adolescents and 9.2% in adults). In ALSPAC, the variables with the highest amount of missing data were alcohol intake (19.5%) in adolescents and smoking in adults (28.6%). We used multivariate imputation with chained equations to impute missing covariates in both Malawi and ALSPAC [40]. Imputation models were performed separately for adolescents and adults and for females and males. The comparison between original and imputed data is presented in Additional file 1: Tables S2 and S3.

### Sensitivity analysis

Considering physical activity was not available in the UK cohort and marital status and parity were not available for UK adolescents, we performed sensitivity analysis excluding these confounders from the associations in the Malawi study.

## Results

Socioeconomic, demographic, behaviour and health characteristics of adolescents and adults in both cohorts are described in Tables 1 and 2. The Malawian cohort was more ethnically mixed (Chewa (35.5%) was the most prevalent ethnicity, followed by Ngoni (19.4%), Lomwe (13.3%) and Tumbuka (13.1%)) than the UK cohort, in which the majority (95%) of participants reported being White/British. Parity was higher in Malawian women and both Malawian women and men were less likely to smoke and consume alcohol than their UK counterparts.

**Table 1:** Characteristics of Malawian urban adolescents and adults according to sex

	Adolescents		Adults	
	Females ***n*** = 774 (62.0%)	Males ***n*** = 474 (38.0%)	Females ***n*** = 1487 (65.3%)	Males ***n*** = 790 (34.7%)
**Age—mean (SD)**	18.7 (0.5)	18.7 (0.5)	47.8 (5.8)	53.4 (7.8)
**Ethnicity—*N*(%)**				
Chewa	285 (36.8)	171 (35.9)	513 (34.5)	282 (35.7)
Tumbuka	104 (13.4)	64 (13.5)	197 (13.3)	98 (12.4)
Ngoni	139 (18.0)	91 (19.3)	317 (21.3)	137 (17.3)
Yao	57 (7.4)	28 (5.9)	118 (7.9)	71 (9.0)
Lomwe	116 (15.0)	87 (18.4)	170 (11.4)	96 (12.2)
Nkonde	10 (1.3)	6 (1.3)	21 (1.4)	12 (1.5)
Other	63 (8.1)	27 (5.7)	151 (10.2)	94 (11.9)
**Education—*N*(%)**				
No formal	10 (1.3)	2 (0.4)	149 (10.0)	23 (2.9)
Primary (1–5 years)	32 (4.1)	10 (2.1)	219 (14.7)	61 (7.7)
Primary (6–8 years)	121 (15.6)	65 (13.8)	492 (33.1)	187 (23.7)
Secondary (9–12 years)	549 (70.9)	368 (77.6)	435 (29.3)	325 (41.1)
Tertiary (12+ years)	62 (8.0)	29 (6.1)	192 (12.9)	194 (24.6)
**Quintiles of household assets score—median (IQR)**				
1 (most deprived)	62.5 (49.0)	70.5 (41.5)	70.5 (41)	70.5 (43)
2	205.5 (35.0)	205.5 (12)	205.5 (51.5)	205.5 (43)
3	325.5 (196.5)	325.5 (145)	325.5 (192)	325.5 (185)
4	520.5 (27.0)	520.5 (35)	524.7 (35)	525.5 (35)
5 (least deprived)	2520.5 (35.0)	2520.5 (35)	2523.3 (35)	2535.5 (35))
**Marital status—*N*(%)**				
Never married	626 (80.9)	471 (99.4)	21 (1.4)	15 (1.9)
Married	134 (17.3)	3 (0.6)	1048 (70.5)	717 (90.8)
Widowed	2 (0.3)	0	277 (18.6)	28 (3.5)
Divorced	12 (1.5)	0	141 (9.5)	30 (3.8)
**Parity—*N*(%)**				
0	594 (76.7)	NA	19 (1.3)	NA
1	167 (21.6)	NA	55 (3.7)	NA
2	9 (1.2)	NA	103 (6.9)	NA
3	1 (0.1)	NA	154 (10.4)	NA
4+	3 (0.4)	NA	1156 (77.7)	NA
**Smoking status—*N*(%)**				
Non-smoker	772 (99.8)	443 (93.5)	1481 (99.6)	644 (81.5)
Former smoker	1 (0.1)	20 (4.2)	4 (0.3)	72 (9.1)
Current smoker	1 (0.1)	11 (2.3)	2 (0.1)	74 (9.4)
**Alcohol intake—*N*(%)**				
Never	737 (95.2)	359 (75.7)	1419 (95.4)	561 (71.0)
< 1 day/month	26 (3.4)	47 (9.9)	35 (2.4)	62 (7.9)
1–3 days/month	6 (0.8)	43 (9.1)	23 (1.5)	73 (9.2)
1–4 days/week	4 (0.5)	18 (3.8)	6 (0.4)	68 (8.6)
5+ days/week	1 (0.1)	7 (1.5)	4 (0.3)	26 (3.3)
**Physical activity level**^**a**^**—*N*(%)**				
Low	4 (0.5)	1 (0.2)	24 (1.6)	79 (10.0)
Moderate	14 (1.8)	31 (6.5)	79 (5.3)	175 (22.1)
High	756 (97.7)	442 (93.3)	1384 (93.1)	536 (67.9)
**Lipid-lowering medication—*N*(%)**				
No raised cholesterol	774 (100.0)	474 (100.0)	1470 (98.9)	781 (98.9)
Raised cholesterol, no medication	0	0	12 (0.8)	8 (1.0)
Raised cholesterol, taking medication	0	0	5 (0.3)	1 (0.1)
**HIV status—*N*(%)**				
HIV negative	695 (97.3)	389 (98.7)	1075 (78.1)	582 (84.1)
HIV positive	19 (2.7)	5 (1.3)	301 (21.9)	110 (15.9)

Adolescent males and females are aged 18 to 19 years, adult females are aged 40 to 60 years, and adult males are aged 43 to 70 years

*HIV* human immunodeficiency virus, *IQR* interquartile range, *NA* not applicable, *SD* standard deviation

^a^Level of physical activity based on metabolic equivalent (MET) and categorised based on the International Physical Activity Questionnaire (IPAQ) [41] scoring protocol

**Table 2:** Characteristics of the UK adolescents and adults according to sex. The Avon Longitudinal Study of Parents and Children (ALSPAC), UK

	Adolescents		Adults	
	Females ***n*** = 1657 (51.8%)	Males ***n*** = 1544 (48.2%)	Females ***n*** = 4423 (69.9%)	Males ***n*** = 1900 (30.0%)
**Age—mean (SD)**	17.8 (0.4)	17.8 (0.4)	47.9 (4.4)	53.3 (5.4)
**Ethnicity—*N*(%)**				
White/British	1509 (95.3)	1417 (95.4)	3939 (97.8)	1700 (98.3)
Other	75 (4.7)	68 (4.6)	87 (2.2)	29 (1.7)
**Education—*N*(%)**^a^				
CSE	178 (11.9)	135 (9.5)	404 (10.0)	105 (6.6)
Vocational degree	96 (6.4)	107 (7.6)	302 (7.5)	85 (5.3)
O level (11 years)	508 (33.9)	436 (30.8)	1385 (34.3)	337 (21.1)
A level (12–13 years)	423 (28.2)	441 (31.1)	1180 (29.2)	486 (30.4)
Degree (13+ years)	293 (19.6)	297 (21.0)	768 (19.0)	584 (36.6)
**Marital status—*N*(%)**				
Never married	NA	NA	94 (3.4)	13 (0.7)
Married	NA	NA	2300 (82.2)	1681 (90.9)
Widowed	NA	NA	40 (1.4)	17 (0.9)
Divorced/separated	NA	NA	365 (13.0)	139 (7.5)
**Parity—*N*(%)**				
1	NA	NA	585 (13.2)	NA
2	NA	NA	1432 (32.4)	NA
3	NA	NA	1123 (25.4)	NA
4+	NA	NA	1283 (29.0)	NA
**Smoking status—*N*(%)**				
Never smoker	641 (46.0)	687 (52.5)	1506 (55.7)	919 (50.8)
Former smoker	356 (25.6)	282 (21.5)	951 (35.1)	730 (40.3)
Current smoker	396 (28.4)	340 (26.0)	248 (9.2)	161 (8.9)
**Alcohol intake—*N*(%)**				
Never	24 (1.8)	32 (2.5)	262 (9.5)	74 (4.0)
Monthly or less	392 (29.6)	281 (22.4)	427 (15.4)	136 (7.4)
2–4 times/month	644 (48.7)	574 (45.8)	513 (18.5)	335 (18.3)
2–3 times/week	234 (17.7)	315 (25.1)	944 (33.9)	720 (39.3)
4+ times/week	29 (2.2)	53 (4.2)	634 (22.8)	567 (31.0)
**Lipid-lowering medication—*N*(%)**				
No medication	NA	NA	4334 (98.2)	1774 (95.7)
Taking medication	NA	NA	81 (1.8)	80 (4.3)

Adolescent males and females are aged 16 to 19 years, adult females are aged 34 to 61 years, and adult males are aged 34 to 89 years

*A level* advanced level, *CSE* certificate of secondary education, *IQR* interquartile range, *NA* not assessed, *O level* ordinary level, *SD* standard deviation

^a^Education corresponds to maternal education for UK adolescents

**Fig. 1:**
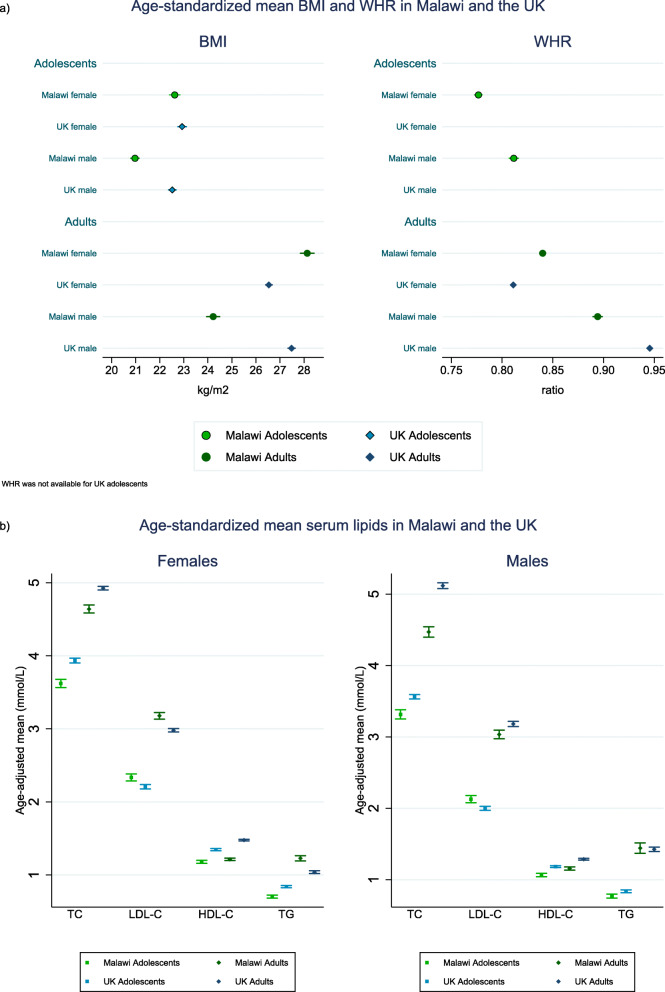
Age-standardised mean BMI and WHR measures (**a**) and serum lipids (**b**) in Malawi and the UK

Malawian female adolescents had similar BMI distributions to UK female adolescents (WHR was not available in the UK cohort), whereas Malawian adult women had higher BMI and WHR than their UK counterparts (Fig. 1a, Additional file 1: Table S4). Malawian males had lower BMI than their UK counterparts in both age groups, and Malawian adult men had lower WHR than UK men. Age-standardised prevalence of overweight, generalised obesity (based on BMI) and central obesity (WHR) followed the same pattern observed for continuous measures. In Malawian adult women, 35% had generalised obesity and 44% had central obesity, whilst the prevalence was 20% and 26%, respectively, in their UK counterparts. About 9% of Malawian adult men had generalised obesity and 46% had central obesity, whereas the prevalence in the UK was 21% and 79%, respectively (Additional file 1: Figure S1).

Overall, Malawian females and males in both age groups had lower TC, higher LDL-C and lower HDL-C than the equivalent UK groups (Fig. 1b; Additional file 1: Table S4). The one exception was LDL-C in adult men, which was lower in the Malawian population. Comparisons of TG distributions between the two populations differed by sex and age, being lower in Malawian, compared with UK adolescents of both sexes, higher in Malawian adult females, and similar in adult males. The binary lipid measures followed the same pattern (Additional file 1: Figure S2). Malawian adolescent females and males and adult females had higher prevalence of any dyslipidaemia than in the UK, whereas the prevalence of any dyslipidaemia was similar in adult males.

The majority of the associations in all groups were linear across the adiposity distributions (Additional file 1: Tables S5 and S6, in mmol/L units, and Figure S3, in standard deviation (SD) units). Exceptions were the associations of BMI with both TC and LDL-C and of WHR with LDL-C in UK adult females, with association magnitudes all being positive and increasing up to the 3rd fifth and then decreasing in strength. As there were no consistent differences across the two countries, all further between-country comparisons assume linearity in both groups.

**Fig. 2:**
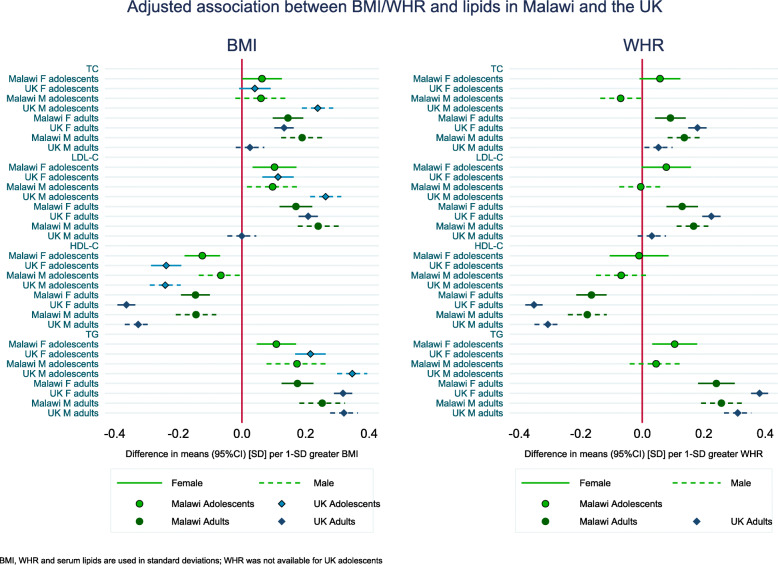
Adjusted association between BMI/WHR and serum lipids in Malawi and the UK

The adjusted associations between BMI/WHR and lipids in adolescents and adults from Malawi and the UK are shown in Fig. 2 (in SD units) and Additional file 1: Table S7 (in mmol/L units). For comparison, age-adjusted associations, presented in Additional file 1: Figure S4, show a similar overall pattern of association between age-only and confounder-adjusted analyses. In general, there were positive associations of BMI and WHR with TC, LDL-C and TG and inverse associations with HDL-C in all four sex and age groups in both Malawi and the UK. The magnitude of positive associations of BMI with TC and LDL-C was similar in Malawian and UK female adolescents and adults, weaker in Malawian compared to UK adolescent males and stronger in Malawian adult males. For example, 1 SD higher BMI was associated with 0.15 mmol/L (95% CI 0.10; 0.19) and 0.16 mmol/L (95% CI 0.14; 0.19) higher LDL-C in Malawi and UK female adults, respectively. In adult males, 1 SD higher BMI was associated with 0.21 mmol/L (95% CI 0.15; 0.27) higher HDL-C in Malawi, whilst no association was observed in the UK (β 0.00, 95% CI − 0.04; 0.04). There was also no evidence for an association in UK adult males between BMI and TC. Inverse associations of BMI with HDL-C and positive associations with TG were stronger in the UK population in all four groups, except for TG in adult males, where the associations were similar; 1 SD higher BMI was associated with 0.23 mmol/L higher TG in both Malawi (95% CI 0.15; 0.30) and the UK (95% CI 0.20; 0.26).

Associations of WHR with lipids were similar to those seen for BMI, with the exception of an inverse association of WHR with TC in Malawian male adolescents. Associations with binary levels of each lipid (high TC, high LDL-C, low HDL-C and high TG) followed similar patterns than observed with continuously measured outcomes (Additional file 1: Table S8). For instance, in female adults, 1 SD higher BMI was associated with 1.4-fold increase in the odds of high LDL-C in both Malawi and the UK, whilst in Malawian male adults, the odds ratio was 1.6 (95% CI 1.4; 1.9) and 1.0 (95% CI 1.0; 1.2) in UK male adults.

There was little evidence of sex differences in the associations between BMI/WHR and lipids in the Malawian cohort. One exception was stronger associations of BMI and WHR with TG in adult males compared with females (Fig. 2, Additional file 1: Table S7). In the UK, associations of BMI with TC, LDL-C and TG were stronger in male compared with female adolescents and associations of BMI and WHR with TC, LDL-C and HDL-C were stronger in female compared with male adults.

## Discussion

Malawian adult females were more adipose and had more adverse lipid profiles than their UK counterparts, whereas the opposite was observed in males. Most associations between BMI/WHR with lipids were similar or weaker in Malawians. However, Malawian adult males had stronger associations of BMI/WHR with TC and LDL-C than their UK counterparts.

Studies in LMIC, including those undertaken in SSA, showed lower cardiovascular disease (CVD) rates than those currently observed in HIC [42–44]. However, there is evidence that mean BMI has steadily increased in SSA populations (and other LMIC), especially in women [21, 45]. This is consistent with our finding in this contemporary study that Malawian women have higher mean BMI and WHR than in the UK, with the opposite for men. As we see largely similar associations of BMI and WHR with lipids between the Malawian and UK population, continued increases in BMI in SSA population will contribute to an increased CVD burden in these poorer settings. When one considers that the ‘obesity epidemic’ has occurred in HIC from the 1970s, when most HIC already had stable modern health systems and very low prevalence of undernutrition, the fact that this is happening now in SSA, when health systems are not equipped to deal with high prevalence of CVD and when undernutrition remains a major health problem, is of concern.

Evidence suggest that African women have more discrepancies than white women regarding perceptions about their own body weight, underestimating their overweight/obesity, with greater disparities observed in the least-educated groups [46, 47]. Whilst there is evidence that they recognise that overweight/obesity is associated with ill-health, African women appear to have more positive perceptions (than white women) about ‘being fat’ as a sign of attractiveness, happiness and lack of diseases, such as HIV and tuberculosis, which may underlie their underestimation of overweight/obesity [46, 47]. A study carried out with black South African adolescents (11–15 years) showed that 25% of the females had a desire to be fatter and that on average those who reported this desire had higher mean BMI than those who were satisfied with their body size or desired to be thinner [48]. Interventions to reduce obesity in SSA are imperative, particularly in women, but these need to consider the complexities surrounding body image perception and attitude, as well as community and cultural perceptions of body size, and possibly need to start in childhood.

A more favourable lipid profile in African ancestry has been shown in many studies [15–18, 49]. This has been linked to polymorphisms in lipid metabolism genes [49] and differences in the activity of lipoprotein lipase, acute insulin response and apolipoprotein CIII concentration [16, 50]. However, in our study, Malawian adult women showed a more adverse lipid profile than their UK counterparts. It is likely that socioeconomic, lifestyle and environmental factors play an important role in these differences, and as energy-dense foods become increasingly easily available in Malawi, any possible genetic benefit will lose any potential protective effect.

Some previous studies in SSA populations report stronger associations between BMI and lipids in men than women [8, 11, 14], whereas we observed similar associations in both sexes in this contemporary Malawian study. Our results of similar or weaker associations in Malawian than UK participants corroborate previous findings of weaker age-adjusted associations of WC with TC and TG in urban Cameroonian women and men than their French counterparts, observed approximately 25 years ago [14]. However, we found additional evidence of a stronger association between BMI/WHR and both TC and LDL-C in Malawian adult men than their UK counterparts, despite being leaner and having similar or lower levels of these lipids. Our results are consistent with contemporary American studies that have reported weaker associations between adiposity and lipids in African American women than their white and Hispanic counterparts [15], including weaker associations between VAT and serum lipids [10, 50], and stronger association between VAT and adverse lipid profile in African American compared with white men [10].

Studies have shown that body composition differs by race/ethnicity, with African origin adults tending to have lower VAT but higher amounts of abdominal subcutaneous adipose tissue (SAT) on average than white European adults, even in African origin women who have evidence of higher overall levels of adiposity [7, 10, 50–52]. These fat distribution differences might contribute to the similar or weaker associations between BMI (and WHR) and lipids in Malawian than UK adult women. Studies suggest that African origin men are more prone to VAT deposition than women [10, 50]. However, given the stronger association of both central and generalised adiposity with TC and LDL-C observed in Malawian than UK men, sex-specific ethnic differences in this association need to be explored further.

### Strengths and limitations

The cross-setting comparison between a low-income, black SSA population and a largely white, high-income European population is a notable strength of this study. It enabled us to explore the extent to which Malawian adolescents and adults have adiposity and lipid distributions comparable to those of a HIC cohort who have a much more advanced stage of economic development and have been exposed to an obesogenic environment for longer. We were also able to compare the magnitude of associations of adiposity with lipids in these two settings with different socioeconomic, demographic and ethnic structures. The use of fasting lipids in both studies is also a further strength.

Given the cross-sectional nature of the study, we cannot assume that the observed associations of adiposity with lipids are causal. However, results from trials of bariatric surgery and Mendelian randomisation studies (which use genetic variants as instrumental variables) suggest causal effects of adiposity on HDL-C and TG, though are less clear for LDL-C, in high-income (mostly white European) populations [5, 53, 54]. Although some of the confounders might not be directly comparable between the two populations (e.g. income and education may have different meanings on associations with BMI or lipids between the two populations), we were able to adjust for a wide range of socioeconomic, demographic and lifestyle factors within each setting. We were unable to adjust for physical activity in the UK cohort and, for UK adolescents, we were also unable to adjust for marital status and parity, as these were not collected at the time that adiposity and lipids were measured. We performed sensitivity analysis excluding these confounders in the Malawi study and the results did not change (Additional file 1: Table S9); therefore, it is unlikely that the residual confounding in the UK cohort has led to the observed differences between Malawi and the UK.

Except for HIV status, there was no missing data in the Malawi study, whereas in the UK cohort, there was missing data on several variables, notably smoking and alcohol consumption. We used multivariate imputation to increase precision in the estimates and reduce selection bias in both studies. This assumes that data are missing at random (i.e. that associations in those with missing data would be similar to those without missing data conditional on characteristics included in our adjusted models). It is possible that violation of this assumption could result in spurious differences in associations between the two populations. However, we would anticipate any such effect to impact the associations with different lipids similarly, whereas that is not what we see. For example, associations of BMI with TC and LDL-C were similar in females from the two populations, whereas Malawian adolescent males had weaker associations than their UK counterparts and the opposite was true for adult men.

We tried to minimise differences between the two cohorts by restricting the comparisons to two groups of similar age. Results for the entire Malawian population are already published [9], and the overall conclusions do not change when comparing the associations with the Malawian urban population without age restriction. We used data from a UK cohort that was collected contemporaneously with that of the Malawi study (2008–2012 in the UK cohort and 2013–2017 in the Malawi study) and used similar methods to measure BMI and lipids. WC and hip circumference were measured differently, at the narrowest part of the abdomen and at the widest part of the buttock, respectively, in Malawi, and in the mid-point between the lower rib and the iliac crest and at the trochanter major, respectively, in the UK. Furthermore, in the UK study, we did not have a measure of WHR in the adolescents. The different measurement protocols could have underestimated central adiposity in Malawian women and men compared to if the same protocol had been used in both studies but would not be expected to bias the associations of WHR with lipids.

It is possible that the family structure (parents and children) in ALSPAC will mean that associations in the adolescents in that cohort are more similar to the adults than in the Malawi cohort. However, we do not think that would have a strong impact on our comparisons. In Malawi, we accounted for multiple individuals in the same age/sex group living in the same household by estimating robust standard errors.

Although most associations were monotonic across the adiposity distributions, there was some evidence of non-linearity in some associations, which means we might have missed some differences between the studies.

## Conclusions

Malawi has one of the poorest populations in the world, yet we have found that urban Malawian female adults (40–60 years) are more adipose and have more adverse lipid profiles than their high-income UK counterparts (34–61 years). Furthermore, most of the associations of BMI and WHR with lipids were similar between the two populations. Thus, our findings highlight the importance of addressing obesity and dyslipidaemia within poor SSA populations such as Malawi to mitigate the oncoming cardiometabolic disease epidemic in this setting. Creation of awareness on individual and community levels of the health risks associated with obesity along with dispelling through educational programmes the positive cultural perception of overweight/obesity is essential for effective interventions.

## Supplementary information

**Additional file 1:** Supplementary methods, tables and figures. **Table S1.** Distribution of covariates in original and imputed data in adolescents in Malawi and the UK, by sex. **Table S2.** Distribution of covariates in original and imputed data in adults in Malawi and the UK, by sex. **Table S3.** Age-standardised means lipids, body mass index (BMI) and waist-hip ratio (WHR) in Malawian and UK adolescents and adults, by sex. **Table S4.** Age-adjusted association between quintiles of BMI and serum lipids in Malawian and UK adolescents and adults, by sex. **Table S5.** Age-adjusted association between quintiles of WHR and serum lipids in Malawian and UK adolescents and adults, by sex. **Table S6.** Adjusted association between BMI/WHR and serum lipids in Malawian and UK adolescents and adults. **Table S7.** Adjusted association between BMI/WHR and dyslipidaemia in Malawian and UK adolescents and adults. **Figure S1.** Age-standardised prevalence of overweight, obesity and central obesity in Malawian and UK adolescents and adults, by sex. **Figure S2.** Age-standardised prevalence of dyslipidaemia in Malawian and UK adolescents and adults, by sex. **Figure S3.** Adjusted association between quintiles of BMI/WHR in adolescents and adults from Malawi (a) and ALSPAC (b). **Figure S4.** Age-adjusted association between BMI/WHR and serum lipids in Malawi and the UK.

## Data Availability

The data that support the finding of this study are available from MEIRU and ALSPAC, but restrictions apply to the availability of these data, which were used under license for the current study, and so are not publicly available. Data are however available from the authors upon reasonable request and with permission of both cohorts.

## Abbreviations

CVD: Cardiovascular disease
IQR: Interquartile range
ALSPAC: The Avon Longitudinal Study of Parents and Children
BMI: Body mass index
CHD: Coronary heart disease
HDL-C: High-density lipoprotein cholesterol
HIC: High-income countries
HIV: Human immunodeficiency virus
LDL-C: Low-density lipoprotein cholesterol
LMIC: Low- and middle-income countries
NCD: Non-communicable diseases
SAT: Subcutaneous adipose tissue
SSA: Sub-Saharan Africa
TC: Total cholesterol
TG: Triglycerides
UK: United Kingdom
VAT: Visceral adipose tissue
WC: Waist circumference
WHR: Waist-hip ratio
