# Supplementary material for: A comparison of the associations between adiposity and lipids in Malawi and the United Kingdom
Source: BMC Med. 2020 Jul 16;18:181. doi: 10.1186/s12916-020-01648-0 (PMC7364601; doi:10.1186/s12916-020-01648-0)
Supplement: Supplementary file 1 — Additional file 1: Supplementary methods, tables and figures. Table S1. Distribution of covariates in original and imputed data in adolescents in Malawi and the UK, by sex. Table S2. Distribution of covariates in original and imputed data in adults in Malawi and the UK, by sex. Table S3. Age-standardised means lipids, body mass index (BMI) and waist-hip ratio (WHR) in Malawian and UK adolescents and adults, by sex. Table S4. Age-adjusted association between quintiles of BMI and serum lipids in Malawian and UK adolescents and adults, by sex. Table S5. Age-adjusted association between quintiles of WHR and serum lipids in Malawian and UK adolescents and adults, by sex. Table S6. Adjusted association between BMI/WHR and serum lipids in Malawian and UK adolescents and adults. Table S7. Adjusted association between BMI/WHR and dyslipidaemia in Malawian and UK adolescents and adults. Figure S1. Age-standardised prevalence of overweight, obesity and central obesity in Malawian and UK adolescents and adults, by sex. Figure S2. Age-standardised prevalence of dyslipidaemia in Malawian and UK adolescents and adults, by sex. Figure S3. Adjusted association between quintiles of BMI/WHR in adolescents and adults from Malawi (a) and ALSPAC (b). Figure S4. Age-adjusted association between BMI/WHR and serum lipids in Malawi and the UK. [file 12916_2020_1648_MOESM1_ESM.pdf]

A comparison of the associations between adiposity and lipids in Malawi and the United Kingdom

Additional file 1: supplementary methods, tables and figures

Supplementary methods

Details of assessment of covariates included in the analyses

In Malawi, ethnicity was assessed in seven groups: Chewa, Tumbuka, Ngoni, Yao, Lomwe, Nkhonde and Other. Household assets score was based on the score of cumulative value, using local costs, of 15 items. The items and respective values are: paraffin lamp 2,000, radio 8,000, mobile phone 10,000, land phone 15,000, table & chair(s) 31,500, bed & mattress 31,000, bicycle 35,000, TV 85,000, electric/gas cooker 30,000, sofa set 125,000, oxcart 200,000, fridge 200,000, motorbike 350,000, car 2,000,000, and cow 150,000. The sum of the score was divided by 1,000 and categorised into quintiles. Educational level was assessed in 7 categories (illiterate, no formal education; literate, no formal education; primary 1-5 years, illiterate; primary 1-5 years, literate; primary 6-8 years; secondary (9-12 years); and tertiary (12 or more years)), and then it was recategorised into: no formal education; primary 1-5 years, primary 6-8 years, secondary, and tertiary. Marital status was assessed as never married, married, widowed or divorced/separated. Parity was considered the number of pregnancies the women ever had, regardless of its outcome. Smoking status comprised regular cigarette smoking (at least weekly), and it was categorised into never smoker, former smoker (stopped more than 6 months ago), and current smoker (smoked in the last 6 months). Alcohol intake in the previous 12 months and its frequency were categorised into: never, less than once a month, 1-3 days per month, 1-4 days per week, and 5 or more days per week. The number of days and frequency of walking, moderate and vigorous physical activity in leisure-time, work and commuting was assessed, and Metabolic Equivalent of Task (MET) was calculated. Physical activity was categorised into low, moderate or high based on the International Physical Activity Questionnaire (IPAQ) scoring protocol (Fogelholm et al., 2006). Low physical activity corresponds to neither moderate nor high criteria; moderate physical activity corresponds to any of the three criteria: (a) 3 days of vigorous activity of at least 20 minutes/day; (b) 5 days of moderate-intensity activity or walking of >30 minutes/day for >10 minutes at a time; or (c) 5 days of any combination of walking, moderate- or vigorous-intensity activities achieving at least 600 MET-minutes/week; high physical activity corresponds to either of two criteria: (a) vigorous-intensity activity on >3 days/week and accumulating at least 1500 MET-minutes/week; or (b) >5 days of any combination of walking, moderate- or vigorous-intensity

activities achieving at least 3000 MET-minutes/week. Use of regular lipid-lowering medication was investigated among those who reported medical diagnosis of raised cholesterol, and it was then recategorised into no raised cholesterol, raised cholesterol not taking medication, and raised cholesterol taking medication. HIV status was also investigated (positive/negative).

In UK adolescents, family socioeconomic data was assessed from questionnaires completed by the mothers during pregnancy and in the first months after delivery. Family income was measured at 33 and 47 months after delivery; a mean family income was calculated, and quintiles were generated. Maternal education was assessed at 32 weeks of gestation in 5 categories (CSE – Certificate of secondary education; Vocational degree; O level – ordinary level; A level – advanced level; and University degree). To note, O level typically represents 11 years of study and marks the end of secondary education; A level is a pre-university qualification requiring an additional 2 years of study and leading to a total of 13 years. Ethnicity was assessed at age 12 years in 5 categories (White, Mixed colour, Asian, Black and Other), and it was recategorised into White and Other because the vast majority (95%) were White. Smoking status and alcohol intake were assessed at the same time as adiposity and lipid measurements. Smoking status was classified into non-smoker, former smoker, and smoker (smoked in the last 30 days). Frequency of alcohol intake was assessed into never, monthly or less, 2-4 times a month, 2-3 times a week, and 4 or more times a week. Use of lipid-lowering medication (yes/no) was investigated among those who reported using any regular medication.

In UK adults, family socioeconomic information was the same as that used for the offspring (see above). Ethnicity was self-reported by the woman and her partner during pregnancy in 7 categories (White, Black Caribbean, Black African, Black Other, Indian, Chinese and other ethnicity); it was recategorised into White/British and Others as 98% were White. All the other covariates were assessed at the same time as assessment of anthropometric and lipids. Marital status was assessed in 9 categories (never married, widowed, divorced, separated, married only once, married for second time, married for third time or more, living as married, and civil partnership), and recategorised into never married, widowed, divorced/separated and married. Parity (number of pregnancies the woman had) was categorised as 1, 2, 3 and 4 or more. Smoking status including cigarette or other smoked tobacco use was categorised into never, former or current smoker. The frequency of alcohol intake was categorised as never, monthly or less, 2-4 times a month, 2-3 times a week, and 4 or more times a week.

Statistical Analysis for dyslipidaemia analysis

Age-standardised prevalence of each dyslipidaemia was described in adolescents and adults in Malawi and the UK, stratified by sex.

Multiple logistic regression analyses were used to examine the association of both BMI and WHR (in standard deviations) with each dyslipidaemia, adjusted initially for age and then for other potential confounders. The same confounders used in the linear regression analyses were used for the logistic regressions. However, for Malawian adolescents, HIV status was removed from the model due to high collinearity, and for ALSPAC adolescents, ethnicity was removed for the same reason.

Differences between the age-standardised prevalence of each dyslipidaemia and the adjusted associations of anthropometric measurements with dyslipidaemias between Malawi and the UK were compared by examining the point estimates and their 95% confidence intervals.

References:

Fogelholm M, Malmberg J, Suni J, Santtila M, Kyrolainen H, Mantysaari M and Oja P. International Physical Activity Questionnaire: Validity against fitness. Med Sci Sports Exerc. 2006;38:753-60.

Table S1. Number of individuals living in the same household in Malawi, by sex and age group

	Females<br>N (%)	Males<br>N (%)
Adolescents		
1	696 (89.9)	432 (91.1)
2	70 (9.0)	39 (8.2)
3+	8 (1.0)	3 (0.6)
Adults		
1	1,280 (86.1)	598 (75.7)
2	202 (13.6)	185 (23.4)
3+	5 (0.3)	7 (0.9)

Table S2. Distribution of covariates in original and imputed data in adolescents in Malawi and the UK, by sex.

			Females		Males	
	% missing	Category	Original<br>% (95% CI)	Imputed<br>% (95% CI)	Original<br>% (95% CI)	Imputed<br>% (95% CI)
**Malawi**						
HIV status	11.2	Positive	2.7 (1.7; 4.1)	2.8 (1.6; 4.0)	1.3 (0.5; 3.0)	1.8 (0.4; 3.3)
**ALSPAC**						
Average income	16.1	Median (IQR)	334.7 (250.9)	322.9 (200.6)	335.2 (255.1)	332.8 (204.6)
Ethnicity	4.1	White	95.3 (94.1; 96.2)	95.2 (94.2; 96.3)	95.4 (94.2; 96.4)	95.4 (94.3; 96.5)
		Other	4.7 (3.8; 5.9)	4.8 (3.7; 5.8)	4.6 (3.6; 5.8)	4.6 (3.5; 5.7)
Education	9.0	CSE	11.9 (10.3; 13.6)	12 (10.3; 13.7)	9.5 (8.1; 11.2)	9.9 (8.3; 11.5)
		Vocational degree	6.4 (5.3; 7.8)	6.6 (5.3; 7.8)	7.6 (6.3; 9.1)	7.5 (6.2; 8.9)
		O-level	33.9 (31.6; 36.4)	33.9 (31.5; 36.3)	30.8 (28.4; 33.2)	31 (28.5; 33.4)
		A-level	28.2 (26.0; 30.6)	28.2 (26.0; 30.5)	31.1 (28.8; 33.6)	30.8 (28.5; 33.2)
		Degree	19.6 (17.6; 21.6)	19.3 (17.3; 21.3)	21 (18.9; 23.2)	20.7 (18.6; 22.8)
Smoking status	15.6	Never	46.0 (43.4; 48.6)	45.8 (43.2; 48.4)	52.5 (49.8; 55.2)	52 (49.3; 54.6)
		Former	25.6 (23.3; 27.9)	25.3 (23.0; 27.6)	21.5 (19.4; 23.9)	21.9 (19.7; 24.2)
		Current	28.4 (26.1; 30.9)	28.9 (26.5; 31.2)	26 (23.7; 28.4)	26.1 (23.7; 28.5)
Alcohol intake	19.5	Never	1.8 (1.2; 2.7)	2.0 (1.2; 2.8)	2.5 (1.8; 3.6)	2.4 (1.6; 3.2)
		Monthly or less	29.6 (27.2; 32.2)	29.9 (27.3; 32.4)	22.4 (20.2; 24.8)	22.5 (20.1; 24.8)
		2-4 times/month	48.7 (46.0; 51.4)	48.1 (45.4; 50.9)	45.7 (43; 48.5)	46.1 (43.3; 48.9)
		2-3 times/week	17.7 (15.7; 19.8)	17.7 (15.8; 19.7)	25.1 (22.8; 27.6)	24.9 (22.5; 27.2)
		4+ times/week	2.2 (1.5; 3.1)	2.2 (1.4; 3.0)	4.2 (3.2; 5.5)	4.2 (3.1; 5.2)

A level – advanced level; CI: confidence interval; CSE – Certificate of secondary education; HIV: human immunodeficiency virus; IQR: interquartile range; O level – ordinary level

Table S3. Distribution of covariates in original and imputed data in adults in Malawi and the UK, by sex.

	% missing	Category	Females		Males	
			Original % (95% CI)	Imputed % (95% CI)	Original % (95% CI)	Imputed % (95% CI)
**Malawi**						
HIV status	9.2%	Positive	21.9 (19.8; 24.1)	21.8 (19.6; 24.0)	15.9 (13.4; 18.8)	16.1 (13.4; 18.9)
**ALSPAC**						
Average income	14.4	Median (IQR)	334.7 (250.9)	322.3 (200.6)	335.3 (255.1)	333.2 (209.6)
Ethnicity	9.0	White	97.8 (97.3; 98.2)	97.7 (97.2; 98.1)	98.3 (97.6; 98.8)	98.1 (97.5; 98.8)
		Other	2.2 (1.8; 2.7)	2.3 (1.9; 2.8)	1.7 (1.2; 2.4)	1.9 (1.2; 2.5)
Education	10.9	CSE	10.0 (9.1; 11)	10.2 (9.2; 11.1)	6.6 (5.5; 7.9)	6.8 (5.6; 8.1)
		Vocational degree	7.5 (6.7; 8.3)	7.5 (6.7; 8.3)	5.3 (4.3; 6.5)	5.5 (4.3; 6.6)
		O-level	34.3 (32.8; 35.8)	34.3 (32.8; 35.8)	21.1 (19.2; 23.2)	21.2 (19.2; 23.2)
		A-level	29.2 (27.8; 30.6)	29.1 (27.7; 30.5)	30.4 (28.2; 32.7)	30.4 (28.1; 32.6)
		Degree	19.0 (17.8; 20.3)	18.9 (17.7; 20.1)	36.6 (34.2; 39.0)	36.2 (33.9; 38.5)
Marital status	26.5	Never married	3.4 (2.8; 4.1)	3.6 (2.9; 4.3)	0.7 (0.4; 1.2)	0.7 (0.3; 1.1)
		Married	82.2 (80.7; 83.5)	81.4 (79.9; 82.9)	90.9 (89.5; 92.1)	90.8 (89.5; 92.2)
		Widowed	1.4 (1.0; 1.9)	1.5 (1.1; 2.0)	0.9 (0.6; 1.5)	0.9 (0.5; 1.4)
		Divorced/ Separated	13.0 (11.8; 14.3)	13.5 (12.1; 14.8)	7.5 (6.4; 8.8)	7.5 (6.3; 8.7)
Smoking status	28.6	Never	55.7 (53.8; 57.5)	54.9 (53; 56.8)	50.8 (48.5; 53.1)	50.8 (48.5; 53.1)
		Former	35.2 (33.4; 37.0)	35.5 (33.8; 37.3)	40.3 (38.1; 42.6)	40.3 (38.0; 42.5)
		Current	9.2 (8.1; 10.3)	9.6 (8.4; 10.7)	8.9 (7.7; 10.3)	8.9 (7.6; 10.2)
Alcohol intake	27.0	Never	9.4 (8.4; 10.6)	9.9 (8.9; 11.0)	4.0 (3.2; 5.0)	4.1 (3.2; 5.0)
		Monthly or less	15.4 (14.1; 16.7)	15.8 (14.4; 17.1)	7.4 (6.3; 8.7)	7.5 (6.2; 8.7)
		2-4 times/month	18.5 (17.1; 19.9)	18.7 (17.2; 20.1)	18.3 (16.6; 20.1)	18.3 (16.5; 20.1)
		2-3 times/week	34.0 (32.2; 35.7)	33.5 (31.8; 35.1)	39.3 (37.1; 41.6)	39.2 (37.0; 41.5)
		4+ times/week	22.8 (21.3; 24.4)	22.2 (20.6; 23.7)	30.9 (28.9; 33.1)	30.9 (28.8; 33.0)
Lipid lowering medication	0.8	No medication	98.2 (97.7; 98.5)	98.2 (97.8; 98.6)	95.7 (94.7; 96.5)	95.7 (94.8; 96.6)
		Taking medication	1.8 (1.5; 2.3)	1.8 (1.4; 2.2)	4.3 (3.5; 5.3)	4.3 (3.4; 5.2)

A level – advanced level; CI: confidence interval; CSE – Certificate of secondary education; HIV: human immunodeficiency virus; IQR: interquartile range; O level – ordinary level

Table S4. Age-standardised means lipids, body mass index (BMI) and waist-hip ratio (WHR) in Malawian and UK adolescents and adults, by sex.

	Females<br>Mean (95% CI)	Males<br>Mean (95% CI)	p-values
**BMI (kg/m^2)**			
Malawi adolescents (N=774) / (N=474)	22.6 (22.4; 22.9)	21.0 (20.8; 21.2)	
UK adolescents (N=1,657) / (N=1,544)	22.9 (22.7; 23.1)	22.5 (22.3; 22.7)	
P-values for difference between sex in Malawi			<0.001
P-values for difference between sex in the UK			<0.001
Malawi adults (N=1,487)/ (N=790)	28.1 (27.8; 28.4)	24.2 (23.9; 24.5)	
UK adults (N=4,423) / (N=1,900)	26.5 (26.4; 26.7)	27.5 (27.3; 27.7)	
P-values for difference between sex in Malawi			<0.001
P-values for difference between sex in the UK			<0.001
**WHR**			
Malawi adolescents (N=774) / (N=474)	0.78 (0.77; 0.78)	0.81 (0.81; 0.82)	
UK adolescents	NA	NA	
P-values for difference between sex in Malawi			<0.001
P-values for difference between sex in the UK			NA
Malawi adults (N=1,487)/ (N=790)	0.84 (0.84; 0.84)	0.89 (0.89; 0.90)	
UK adults (N=4,423) / (N=1,900)	0.81 (0.81; 0.81)	0.95 (0.94; 0.95)	
P-values for difference between sex in Malawi			<0.001
P-values for difference between sex in the UK			<0.001
**TC (mmol/L)**			
Malawi adolescents (N=774) / (N=474)	3.62 (3.57; 3.68)	3.32 (3.25; 3.38)	
UK adolescents (N=1,657) / (N=1,544)	3.94 (3.90; 3.97)	3.56 (3.53; 3.59)	
P-values for difference between sex in Malawi			<0.001
P-values for difference between sex in the UK			<0.001
Malawi adults (N=1,487)/ (N=790)	4.64 (4.59; 4.69)	4.47 (4.40; 4.54)	
UK adults (N=4,423) / (N=1,900)	4.93 (4.90; 4.95)	5.12 (5.08; 5.16)	
P-values for difference between sex in Malawi			<0.001
P-values for difference between sex in the UK			0.019
**LDL-C (mmol/L)**			
Malawi adolescents (N=774) / (N=474)	2.34 (2.29; 2.38)	2.13 (2.08; 2.18)	
UK adolescents (N=1,657) / (N=1,544)	2.21 (2.18; 2.24)	2.00 (1.97; 2.03)	
P-values for difference between sex in Malawi			<0.001
P-values for difference between sex in the UK			<0.001
Malawi adults (N=1,487)/ (N=790)	3.18 (3.13; 3.22)	3.03 (2.97; 3.10)	
UK adults (N=4,423) / (N=1,900)	2.98 (2.96; 3.00)	3.18 (3.15; 3.22)	
P-values for difference between sex in Malawi			<0.001
P-values for difference between sex in the UK			<0.001
**HDL-C (mmol/L)**			
Malawi adolescents (N=774) / (N=474)	1.18 (1.16; 1.20)	1.07 (1.04; 1.09)	
UK adolescents (N=1,657) / (N=1,544)	1.35 (1.33; 1.36)	1.18 (1.17; 1.19)	
P-values for difference between sex in Malawi			<0.001
P-values for difference between sex in the UK			<0.001
Malawi adults (N=1,487)/ (N=790)	1.21 (1.20; 1.23)	1.16 (1.13; 1.18)	
UK adults (N=4,423) / (N=1,900)	1.48 (1.47; 1.49)	1.29 (1.27; 1.30)	
P-values for difference between sex in Malawi			<0.001
P-values for difference between sex in the UK			<0.001
**TG (mmol/L)**			
Malawi adolescents (N=774) / (N=474)	0.70 (0.68; 0.72)	0.77 (0.74; 0.80)	
UK adolescents (N=1,657) / (N=1,544)	0.84 (0.82; 0.86)	0.84 (0.82; 0.86)	
P-values for difference between sex in Malawi			<0.001
P-values for difference between sex in the UK			0.842
Malawi adults (N=1,487)/ (N=790)	1.23 (1.19; 1.26)	1.44 (1.37; 1.52)	
UK adults (N=4,423) / (N=1,900)	1.04 (1.02; 1.06)	1.43 (1.40; 1.46)	
P-values for difference between sex in Malawi			<0.001
P-values for difference between sex in the UK			<0.001

BMI: body mass index; HDL-C: high density lipoprotein-cholesterol; LDL-C: low density lipoprotein-cholesterol; NA: not assessed; TC: total cholesterol; TG: triglycerides; WHR: waist-hip ratio

Table S5. Age-adjusted association between quintiles of BMI and serum lipids in Malawian and UK adolescents and adults, by sex.

Outcome	Quintile	Adolescents				Adults			
		Females		Males		Females		Males	
		Beta (95% CI)	p-value LRT	Beta (95% CI)	p-value LRT	Beta (95% CI)	p-value LRT	Beta (95% CI)	p-value LRT
Malawi									
TC	2nd	0.04 (-0.17; 0.26)	0.815	-0.20 (-0.50; 0.10)	0.289	0.07 (-0.09; 0.23)	0.690	0.24 (0.02; 0.46)	0.084
	3rd	0.03 (-0.18; 0.25)		-0.01 (-0.32; 0.30)		0.17 (0.00; 0.33)		0.38 (0.16; 0.61)	
	4th	0.06 (-0.17; 0.28)		0.13 (-0.16; 0.42)		0.38 (0.21; 0.54)		0.47 (0.24; 0.71)	
	5th	0.25 (0.03; 0.47)		0.03 (-0.28; 0.34)		0.38 (0.21; 0.55)		0.67 (0.44; 0.91)	
LDL	2nd	-0.08 (-0.30; 0.14)	0.553	-0.02 (-0.33; 0.28)	0.757	0.13 (-0.03; 0.30)	0.038	0.29 (0.08; 0.50)	0.006
	3rd	0.02 (-0.20; 0.24)		0.17 (-0.15; 0.48)		0.26 (0.10; 0.43)		0.47 (0.25; 0.69)	
	4th	0.06 (-0.17; 0.29)		0.28 (-0.02; 0.57)		0.51 (0.34; 0.68)		0.59 (0.36; 0.81)	
	5th	0.32 (0.10; 0.55)		0.26 (-0.06; 0.57)		0.49 (0.32; 0.67)		0.88 (0.66; 1.11)	
HDL	2nd	-0.06 (-0.27; 0.15)	1.000	-0.29 (-0.57; 0.00)	0.085	-0.20 (-0.37; -0.04)	0.142	0.04 (-0.18; 0.25)	0.437
	3rd	-0.05 (-0.26; 0.16)		-0.36 (-0.66; -0.06)		-0.27 (-0.43; -0.10)		-0.22 (-0.44; 0.00)	
	4th	-0.15 (-0.37; 0.07)		-0.20 (-0.47; 0.08)		-0.44 (-0.61; -0.28)		-0.35 (-0.57; -0.12)	
	5th	-0.30 (-0.51; -0.08)		-0.34 (-0.64; -0.04)		-0.53 (-0.70; -0.36)		-0.42 (-0.65; -0.19)	
TG	2nd	0.00 (-0.18; 0.18)	0.825	-0.03 (-0.31; 0.24)	0.530	0.16 (-0.01; 0.34)	1.000	0.05 (-0.20; 0.30)	0.011
	3rd	0.08 (-0.10; 0.26)		0.24 (-0.05; 0.53)		0.28 (0.10; 0.45)		0.27 (0.01; 0.52)	
	4th	0.14 (-0.05; 0.33)		0.16 (-0.11; 0.43)		0.47 (0.29; 0.65)		0.62 (0.35; 0.89)	
	5th	0.30 (0.12; 0.49)		0.41 (0.12; 0.70)		0.55 (0.37; 0.73)		0.77 (0.50; 1.03)	
UK									
TC	2nd	0.24 (0.05; 0.44)	0.004	0.03 (-0.16; 0.22)	1.000	0.10 (-0.02; 0.21)	0.013	0.25 (0.08; 0.42)	0.004
	3rd	0.36 (0.17; 0.54)		0.11 (-0.08; 0.30)		0.21 (0.09; 0.33)		0.23 (0.07; 0.39)	
	4th	0.28 (0.09; 0.47)		0.39 (0.20; 0.58)		0.32 (0.20; 0.44)		0.25 (0.10; 0.41)	
	5th	0.29 (0.10; 0.49)		0.61 (0.42; 0.80)		0.36 (0.24; 0.49)		0.16 (0.00; 0.32)	
LDL	2nd	0.28 (0.09; 0.47)	0.001	0.04 (-0.14; 0.23)	1.000	0.19 (0.08; 0.30)	<0.001	0.17 (0.00; 0.34)	0.125
	3rd	0.44 (0.25; 0.62)		0.14 (-0.05; 0.33)		0.37 (0.25; 0.49)		0.12 (-0.05; 0.28)	
	4th	0.44 (0.25; 0.63)		0.42 (0.23; 0.61)		0.51 (0.39; 0.63)		0.14 (-0.01; 0.30)	
	5th	0.52 (0.32; 0.72)		0.72 (0.53; 0.91)		0.59 (0.47; 0.72)		0.04 (-0.12; 0.21)	
HDL	2nd	-0.10 (-0.28; 0.08)	1.000	-0.04 (-0.22; 0.14)	1.000	-0.28 (-0.39; -0.18)	0.881	-0.19 (-0.34; -0.03)	0.018

	3rd	-0.23 (-0.41; -0.05)		-0.10 (-0.28; 0.09)		-0.46 (-0.57; -0.35)		-0.57 (-0.72; -0.42)	
	4th	-0.39 (-0.57; -0.21)		-0.19 (-0.38; -0.01)		-0.77 (-0.88; -0.66)		-0.61 (-0.76; -0.46)	
	5th	-0.67 (-0.86; -0.49)		-0.60 (-0.78; -0.41)		-1.05 (-1.16; -0.93)		-0.99 (-1.14; -0.84)	
TG	2nd	0.14 (-0.04; 0.33)	1.000	0.04 (-0.13; 0.21)	1.000	0.13 (0.03; 0.24)	<0.001	0.31 (0.15; 0.47)	<0.001
	3rd	0.23 (0.05; 0.41)		0.07 (-0.10; 0.24)		0.24 (0.13; 0.35)		0.58 (0.42; 0.74)	
	4th	0.24 (0.06; 0.43)		0.30 (0.13; 0.47)		0.61 (0.50; 0.71)		0.63 (0.48; 0.79)	
	5th	0.57 (0.38; 0.77)		0.76 (0.59; 0.93)		0.87 (0.76; 0.99)		1.02 (0.87; 1.18)	

1st quintile was considered the reference category

BMI: body mass index; HDL-C: high density lipoprotein-cholesterol; LDL-C: low density lipoprotein-cholesterol; LRT: likelihood ratio test; TC: total cholesterol; TG: triglycerides

Table S6. Age-adjusted association between quintiles of WHR and serum lipids in Malawian and UK adolescents and adults, by sex.

Outcome	Quintile	Adolescents				Adults			
		Females		Males		Females		Males	
		Beta (95% CI)	p-value LRT	Beta (95% CI)	p-value LRT	Beta (95% CI)	p-value LRT	Beta (95% CI)	p-value LRT
Malawi									
TC	2nd	-0.03 (-0.24; 0.19)	0.278	0.16 (-0.14; 0.47)	0.958	0.06 (-0.09; 0.22)	0.229	0.10 (-0.16; 0.35)	0.493
	3rd	0.10 (-0.13; 0.32)		0.12 (-0.18; 0.42)		0.12 (-0.04; 0.27)		0.12 (-0.11; 0.36)	
	4th	-0.10 (-0.32; 0.13)		0.03 (-0.27; 0.33)		0.23 (0.07; 0.39)		0.24 (0.01; 0.48)	
	5th	0.18 (-0.06; 0.41)		-0.07 (-0.37; 0.22)		0.28 (0.11; 0.45)		0.53 (0.31; 0.75)	
LDL	2nd	0.01 (-0.21; 0.24)	0.385	0.21 (-0.10; 0.52)	0.441	0.11 (-0.05; 0.27)	0.030	0.07 (-0.17; 0.32)	0.456
	3rd	0.09 (-0.14; 0.32)		0.23 (-0.08; 0.54)		0.17 (0.01; 0.33)		0.15 (-0.08; 0.39)	
	4th	-0.05 (-0.28; 0.19)		0.17 (-0.14; 0.48)		0.34 (0.17; 0.50)		0.37 (0.14; 0.60)	
	5th	0.27 (0.03; 0.51)		0.15 (-0.15; 0.46)		0.43 (0.25; 0.60)		0.60 (0.38; 0.82)	
HDL	2nd	-0.04 (-0.25; 0.17)	0.125	0.31 (0.02; 0.60)	0.002	-0.08 (-0.23; 0.08)	0.371	-0.07 (-0.31; 0.17)	0.010
	3rd	-0.02 (-0.24; 0.20)		0.19 (-0.10; 0.47)		-0.30 (-0.46; -0.14)		-0.27 (-0.50; -0.05)	
	4th	-0.21 (-0.43; 0.01)		0.11 (-0.17; 0.39)		-0.37 (-0.53; -0.21)		-0.42 (-0.64; -0.19)	
	5th	-0.18 (-0.41; 0.05)		-0.24 (-0.52; 0.04)		-0.51 (-0.69; -0.34)		-0.59 (-0.80; -0.38)	
TG	2nd	-0.02 (-0.20; 0.17)	0.020	-0.11 (-0.40; 0.17)	0.143	0.09 (-0.07; 0.25)	0.005	0.05 (-0.23; 0.33)	0.152
	3rd	0.04 (-0.14; 0.23)		0.07 (-0.21; 0.35)		0.39 (0.22; 0.55)		0.24 (-0.02; 0.51)	
	4th	0.02 (-0.17; 0.21)		0.16 (-0.12; 0.44)		0.59 (0.43; 0.76)		0.53 (0.27; 0.79)	
	5th	0.32 (0.12; 0.52)		0.19 (-0.09; 0.47)		0.71 (0.53; 0.89)		0.84 (0.59; 1.09)	
UK									
TC	2nd	WHR was not assessed in adolescents				0.07 (-0.04; 0.19)	0.010	0.09 (-0.08; 0.25)	0.111
	3rd					0.30 (0.18; 0.42)		0.21 (0.05; 0.38)	
	4th					0.39 (0.27; 0.51)		0.20 (0.04; 0.36)	
	5th					0.45 (0.33; 0.58)		0.12 (-0.05; 0.28)	
LDL	2nd					0.12 (0.01; 0.24)	0.010	0.15 (-0.02; 0.31)	0.041
	3rd					0.37 (0.25; 0.48)		0.23 (0.07; 0.39)	
	4th					0.55 (0.43; 0.67)		0.18 (0.01; 0.34)	
	5th					0.62 (0.50; 0.74)		0.08 (-0.09; 0.25)	
HDL	2nd					-0.18 (-0.28; -0.07)	1.000	-0.43 (-0.58; -0.27)	0.324

	3rd	-0.34 (-0.45; -0.22)		-0.54 (-0.69; -0.39)	
	4th	-0.68 (-0.79; -0.56)		-0.75 (-0.90; -0.60)	
	5th	-0.89 (-1.01; -0.78)		-0.96 (-1.12; -0.81)	
TG	2nd	0.12 (0.02; 0.22)	1.000	0.28 (0.12; 0.44)	0.680
	3rd	0.33 (0.22; 0.43)		0.46 (0.30; 0.62)	
	4th	0.57 (0.46; 0.68)		0.62 (0.46; 0.78)	
	5th	0.91 (0.80; 1.02)		0.89 (0.73; 1.05)	

1st quintile was considered the reference category

HDL-C: high density lipoprotein-cholesterol; LDL-C: low density lipoprotein-cholesterol; LRT: likelihood ratio test; TC: total cholesterol; TG: triglycerides; WHR: waist-hip ratio

Table S7. Adjusted association between BMI/WHR and serum lipids in Malawian and UK adolescents and adults

	Adjusted difference in outcome per 1 SD higher BMI (95% CI)	Adjusted difference in outcome per 1 SD higher WHR (95% CI)
**Difference in mean TC (mmol/L)**		
Malawi female adolescents (N=774)	0.05 (0.00; 0.11)	0.05 (-0.01; 0.10)
UK female adolescents (N=1,657)	0.03 (0.00; 0.06)	WHR not measured
Malawi male adolescents (N=474)	0.04 (-0.01; 0.10)	-0.05 (-0.10; 0.00)
UK male adolescents (N=1,544)	0.15 (0.12; 0.18)	WHR not measured
P-values for difference between sex in Malawi	0.662	0.003
P-values for difference between sex in the UK	<0.001	NA
Malawi female adults (N=1,487)	0.15 (0.10; 0.20)	0.10 (0.05; 0.15)
UK female adults (N=3,847)	0.11 (0.08; 0.13)	0.15 (0.12; 0.17)
Malawi male adults (N=790)	0.20 (0.13; 0.27)	0.15 (0.09; 0.21)
UK male adults (N=1,739)	0.02 (-0.02; 0.07)	0.05 (0.01; 0.09)
P-values for difference between sex in Malawi	0.417	0.193
P-values for difference between sex in the UK	<0.001	<0.001
**Difference in mean LDL-C (mmol/L)**		
Malawi female adolescents (N=774)	0.07 (0.02; 0.12)	0.05 (0.00; 0.11)
UK female adolescents (N=1,657)	0.07 (0.04; 0.10)	NA
Malawi male adolescents (N=474)	0.05 (0.01; 0.10)	0.00 (-0.04; 0.04)
UK male adolescents (N=1,544)	0.15 (0.12; 0.18)	NA
P-values for difference between sex in Malawi	0.507	0.054
P-values for difference between sex in the UK	<0.001	NA
Malawi female adults (N=1,487)	0.15 (0.10; 0.19)	0.12 (0.07; 0.16)
UK female adults (N=3,847)	0.16 (0.14; 0.19)	0.17 (0.15; 0.20)
Malawi male adults (N=790)	0.21 (0.15; 0.27)	0.15 (0.10; 0.20)
UK male adults (N=1,739)	0.00 (-0.04; 0.04)	0.03 (-0.01; 0.07)
P-values for difference between sex in Malawi	0.126	0.203
P-values for difference between sex in the UK	<0.001	<0.001
**Difference in mean HDL-C (mmol/L)**		
Malawi female adolescents (N=774)	-0.04 (-0.06; -0.02)	0.00 (-0.03; 0.03)
UK female adolescents (N=1,657)	-0.07 (-0.09; -0.06)	NA
Malawi male adolescents (N=474)	-0.02 (-0.04; 0.00)	-0.02 (-0.04; 0.00)
UK male adolescents (N=1,544)	-0.06 (-0.07; -0.05)	NA
P-values for difference between sex in Malawi	0.238	0.258
P-values for difference between sex in the UK	0.141	NA
Malawi female adults (N=1,487)	-0.05 (-0.06; -0.03)	-0.06 (-0.07; -0.04)
UK female adults (N=3,847)	-0.14 (-0.15; -0.13)	-0.13 (-0.15; -0.12)
Malawi male adults (N=790)	-0.05 (-0.08; -0.03)	-0.06 (-0.09; -0.04)
UK male adults (N=1,739)	-0.08 (-0.09; -0.07)	-0.08 (-0.09; -0.07)
P-values for difference between sex in Malawi	0.932	0.565
P-values for difference between sex in the UK	<0.001	<0.001
**Difference in mean TG (mmol/L)**		
Malawi female adolescents (N=774)	0.04 (0.02; 0.06)	0.04 (0.01; 0.06)
UK female adolescents (N=1,657)	0.07 (0.06; 0.09)	NA
Malawi male adolescents (N=474)	0.06 (0.03; 0.09)	0.02 (-0.01; 0.05)
UK male adolescents (N=1,544)	0.14 (0.12; 0.15)	NA
P-values for difference between sex in Malawi	0.290	0.295

P-values for difference between sex in the UK	<0.001	NA
Malawi female adults (N=1,487)	0.10 (0.07; 0.14)	0.15 (0.11; 0.18)
UK female adults (N=3,847)	0.19 (0.17; 0.21)	0.24 (0.22; 0.26)
Malawi male adults (N=790)	0.23 (0.16; 0.29)	0.23 (0.17; 0.29)
UK male adults (N=1,739)	0.23 (0.20; 0.26)	0.22 (0.19; 0.26)
P-values for difference between sex in Malawi	0.010	0.031
P-values for difference between sex in the UK	0.053	0.248

BMI: body mass index; HDL-C: high density lipoprotein-cholesterol; LDL-C: low density lipoprotein-cholesterol; NA: not assessed; TC: total cholesterol; TG: triglycerides; WHR: waist-hip ratio

Malawi adolescents: Adjusted for age, ethnicity, education, household assets score, marital status, parity (females), smoking status, alcohol intake, physical activity, and HIV status.

UK Adolescents: Adjusted for age, ethnicity, maternal education, smoking status, and alcohol intake.

Malawi adults: Adjusted for age, ethnicity, education, household assets score, marital status, parity (females), use of lipid-lowering medication, smoking status, alcohol intake, physical activity, and HIV status.

UK adults: Adjusted for age, ethnicity, education, family income, marital status, parity (females), use of lipid-lowering medication, smoking status, and alcohol intake.

Table S8. Adjusted association between BMI/WHR and dyslipidaemia in Malawian and UK adolescents and adults

	Adjusted difference in outcome per 1 SD higher BMI (95% CI)	Adjusted difference in outcome per 1 SD higher WHR (95% CI)
**High TC**		
Malawi female adolescents (N=774)	1.2 (0.9; 1.7)	1.0 (0.7; 1.4)
UK female adolescents (N=1,657)	1.0 (0.8; 1.3)	NA
Malawi male adolescents (N=474)	1.4 (1.0; 2.0)	1.4 (1.1; 1.7)
UK male adolescents (N=1,544)	1.6 (1.2; 2.4)	NA
P-values for difference between sex in Malawi	0.471	0.164
P-values for difference between sex in the UK	0.018	NA
Malawi female adults (N=1,487)	1.3 (1.2; 1.5)	1.2 (1.1; 1.4)
UK female adults (N=3,847)	1.2 (1.1; 1.3)	1.4 (1.3; 1.5)
Malawi male adults (N=790)	1.5 (1.3; 1.8)	1.4 (1.2; 1.7)
UK male adults (N=1,739)	1.1 (1.0; 1.2)	1.1 (1.0; 1.3)
P-values for difference between sex in Malawi	0.271	0.179
P-values for difference between sex in the UK	0.005	<0.001
**High LDL-C**		
Malawi female adolescents (N=774)	1.1 (0.9; 1.5)	1.4 (1.1; 1.7)
UK female adolescents (N=1,657)	1.3 (1.1; 1.6)	NA
Malawi male adolescents (N=474)	1.2 (0.8; 1.7)	1.0 (0.7; 1.4)
UK male adolescents (N=1,544)	1.6 (1.2; 2.2)	NA
P-values for difference between sex in Malawi	0.998	0.111
P-values for difference between sex in the UK	0.199	NA
Malawi female adults (N=1,487)	1.4 (1.3; 1.6)	1.3 (1.1; 1.4)
UK female adults (N=3,847)	1.4 (1.3; 1.5)	1.5 (1.4; 1.7)
Malawi male adults (N=790)	1.6 (1.4; 1.9)	1.6 (1.4; 1.9)
UK male adults (N=1,739)	1.0 (1.0; 1.2)	1.1 (1.0; 1.3)
P-values for difference between sex in Malawi	0.455	0.010
P-values for difference between sex in the UK	<0.001	<0.001
**Low HDL-C**		
Malawi female adolescents (N=774)	1.3 (1.1; 1.5)	1.2 (1.0; 1.4)
UK female adolescents (N=1,657)	1.6 (1.4; 1.9)	NA
Malawi male adolescents (N=474)	1.3 (1.0; 1.6)	1.1 (0.9; 1.3)
UK male adolescents (N=1,544)	1.6 (1.4; 1.8)	NA
P-values for difference between sex in Malawi	0.892	0.808
P-values for difference between sex in the UK	0.914	NA
Malawi female adults (N=1,487)	1.3 (1.2; 1.5)	1.5 (1.3; 1.7)
UK female adults (N=3,847)	1.9 (1.7; 2.1)	2.2 (2.0; 2.5)
Malawi male adults (N=790)	1.2 (1.0; 1.4)	1.3 (1.1; 1.5)
UK male adults (N=1,739)	2.0 (1.8; 2.3)	1.7 (1.5; 2.0)
P-values for difference between sex in Malawi	0.425	0.228
P-values for difference between sex in the UK	0.306	0.007
**High TG**		
Malawi female adolescents (N=774)	1.6 (0.8; 3.3)	1.7 (1.1; 2.6)
UK female adolescents (N=1,657)	1.7 (1.4; 2.1)	NA
Malawi male adolescents (N=474)	5.4 (1.8; 15.7)	1.5 (1.0; 2.2)
UK male adolescents (N=1,544)	2.4 (1.9; 3.0)	NA
P-values for difference between sex in Malawi	0.021	0.915

P-values for difference between sex in the UK	0.031	NA
Malawi female adults (N=1,487)	1.5 (1.3; 1.7)	1.9 (1.6; 2.2)
UK female adults (N=3,847)	1.9 (1.7; 2.0)	2.4 (2.2; 2.7)
Malawi male adults (N=790)	1.8 (1.5; 2.1)	1.9 (1.6; 2.3)
UK male adults (N=1,739)	1.9 (1.7; 2.1)	1.8 (1.6; 2.0)
P-values for difference between sex in Malawi	0.274	0.967
P-values for difference between sex in the UK	0.652	<0.001

BMI: body mass index; HDL-C: high density lipoprotein-cholesterol; LDL-C: low density lipoprotein-cholesterol; NA: not assessed; TC: total cholesterol; TG: triglycerides; WHR: waist-hip ratio

Malawi adolescents: Adjusted for age, ethnicity, education, household assets score, marital status, parity (females), smoking status, alcohol intake, physical activity.

UK adolescents: Adjusted for age, maternal education, smoking status, and alcohol intake.

Malawi adults: Adjusted for age, ethnicity, education, household assets score, marital status, parity (females), use of lipid-lowering medication, smoking status, alcohol intake, physical activity, and HIV status.

UK adults: Adjusted for age, ethnicity, education, family income, marital status, parity (females), use of lipid-lowering medication, smoking status, and alcohol intake.

Table S9. Adjusted association between BMI/WHR and serum lipids in Malawian and UK adolescents and adults

	**Adjusted difference in outcome per 1 SD higher BMI (95% CI)**	**Adjusted difference in outcome per 1 SD higher WHR (95% CI)**
**Difference in mean TC (mmol/L)**		
Malawi female adolescents (N=774)	0.06 (0.00; 0.13)	0.06 (-0.01; 0.12)
Malawi male adolescents (N=474)	0.06 (-0.02; 0.14)	-0.07 (-0.13; 0.00)
P-values for difference between sex in Malawi	0.759	0.004
Malawi female adults (N=1,487)	0.14 (0.10; 0.19)	0.09 (0.04; 0.14)
Malawi male adults (N=790)	0.19 (0.12; 0.26)	0.14 (0.08; 0.19)
P-values for difference between sex in Malawi	0.393	0.208
**Difference in mean LDL-C (mmol/L)**		
Malawi female adolescents (N=774)	0.10 (0.03; 0.17)	0.08 (0.00; 0.16)
Malawi male adolescents (N=474)	0.10 (0.02; 0.18)	0.00 (-0.07; 0.07)
P-values for difference between sex in Malawi	0.760	0.070
Malawi female adults (N=1,487)	0.17 (0.12; 0.22)	0.13 (0.08; 0.18)
Malawi male adults (N=790)	0.24 (0.18; 0.31)	0.17 (0.11; 0.22)
P-values for difference between sex in Malawi	0.100	0.227
**Difference in mean HDL-C (mmol/L)**		
Malawi female adolescents (N=774)	-0.12 (-0.18; -0.07)	-0.01 (-0.11; 0.08)
Malawi male adolescents (N=474)	-0.07 (-0.14; 0.00)	-0.07 (-0.15; 0.01)
P-values for difference between sex in Malawi	0.359	0.285
Malawi female adults (N=1,487)	-0.15 (-0.19; -0.10)	-0.17 (-0.22; -0.12)
Malawi male adults (N=790)	-0.15 (-0.21; -0.08)	-0.18 (-0.24; -0.12)
P-values for difference between sex in Malawi	0.914	0.683
**Difference in mean TG (mmol/L)**		
Malawi female adolescents (N=774)	0.10 (0.04; 0.17)	0.10 (0.02; 0.17)
Malawi male adolescents (N=474)	0.17 (0.08; 0.27)	0.04 (-0.04; 0.13)
P-values for difference between sex in Malawi	0.214	0.335
Malawi female adults (N=1,487)	0.18 (0.13; 0.23)	0.24 (0.18; 0.30)
Malawi male adults (N=790)	0.25 (0.18; 0.32)	0.26 (0.19; 0.32)
P-values for difference between sex in Malawi	0.218	0.910

BMI: body mass index; HDL-C: high density lipoprotein-cholesterol; LDL-C: low density lipoprotein-cholesterol; NA: not assessed; TC: total cholesterol; TG: triglycerides; WHR: waist-hip ratio

Malawi adolescents: Adjusted for age, ethnicity, education, household assets score, smoking status, alcohol intake, and HIV status.

Malawi adults: Adjusted for age, ethnicity, education, household assets score, marital status, parity (females), use of lipid-lowering medication, smoking status, alcohol intake, and HIV status.

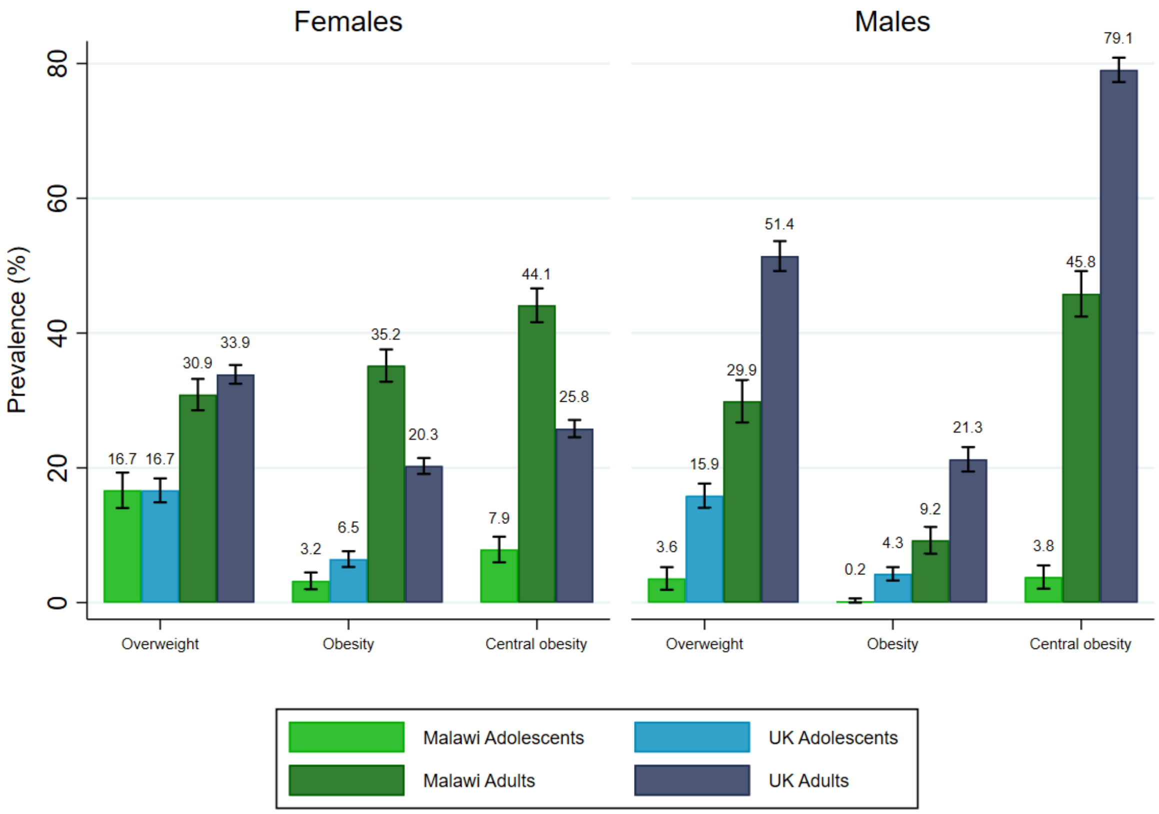

Figure S1. Age-standardised prevalence of overweight, obesity and central obesity in Malawian and UK adolescents and adults, by sex.

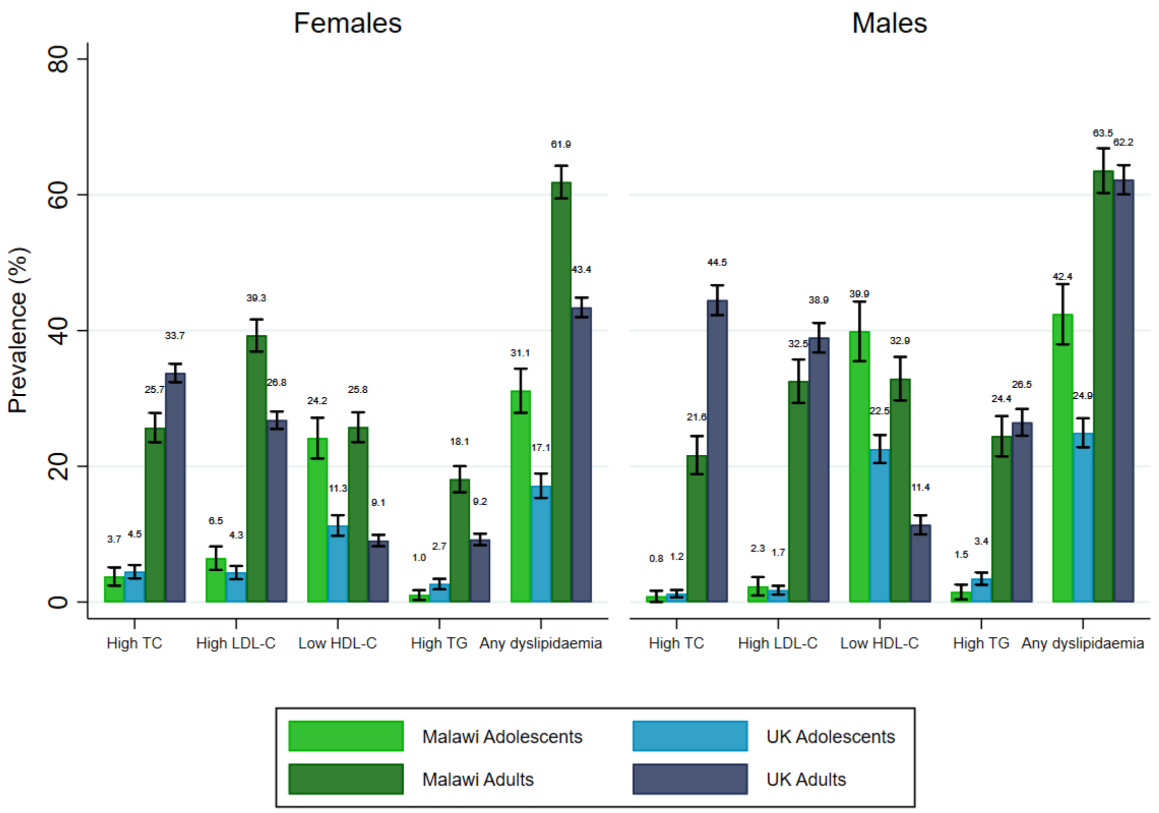

Figure S2. Age-standardised prevalence of dyslipidaemia in Malawian and UK adolescents and adults, by sex.

HDL-C: high density lipoprotein-cholesterol; LDL-C: low density lipoprotein-cholesterol; TC: total cholesterol; TG: triglycerides

a)

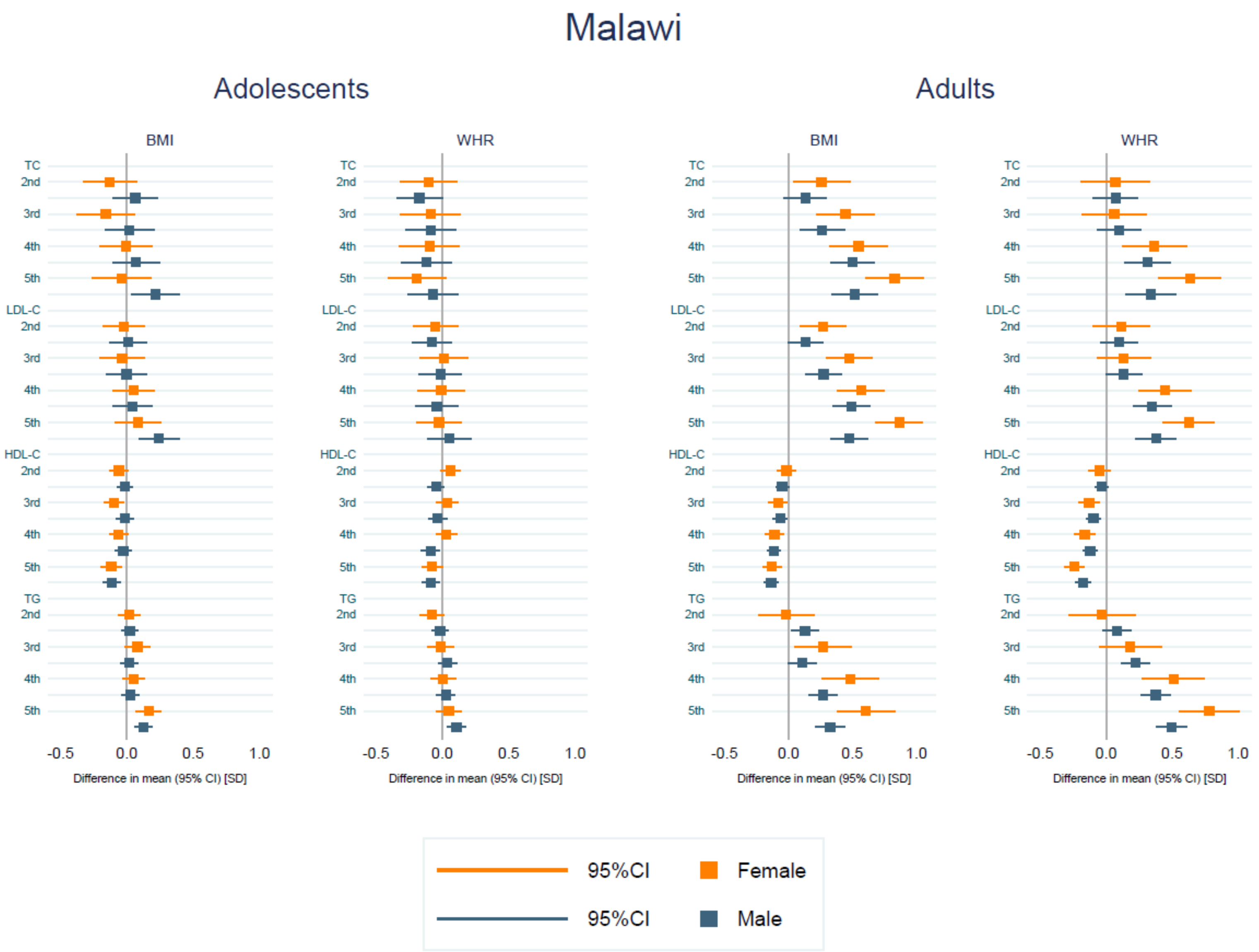

Malawi
Adolescents
Adults
BMI
WHR
TC
2nd
3rd
4th
5th
LDL-C
HDL-C
TG
-0.5
0.0
0.5
1.0
Difference in mean (95% CI) [SD]
95%CI
Female
Male

b)

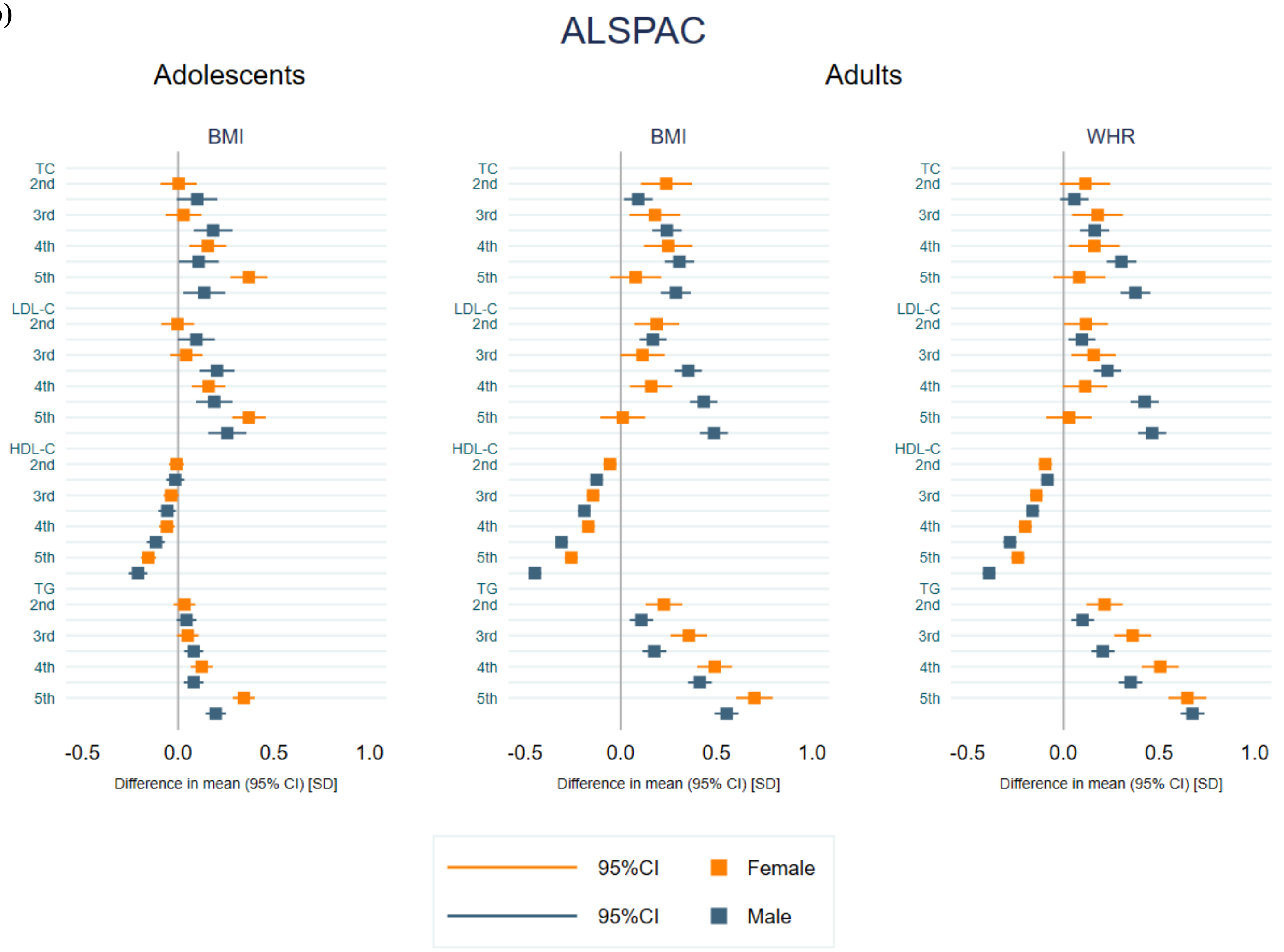

Figure S3. Adjusted association between quintiles of BMI/WHR in adolescents and adults from Malawi (a) and ALSPAC (b).

BMI: body mass index; HDL-C: high density lipoprotein-cholesterol; LDL-C: low density lipoprotein-cholesterol; NA: not assessed; TC: total cholesterol; TG: triglycerides; WHR: waist-hip ratio

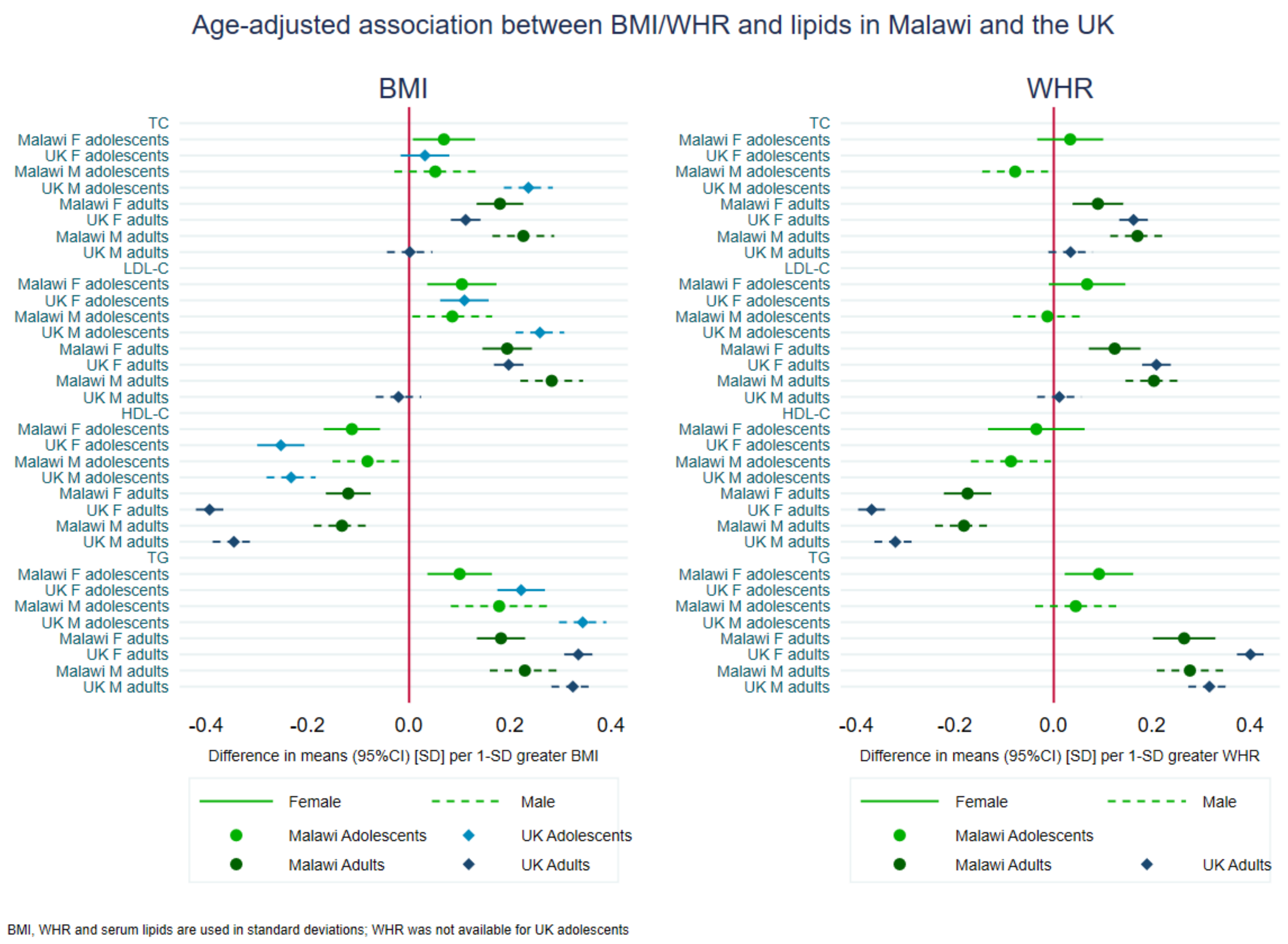

Figure S4. Age-adjusted association between BMI/WHR and serum lipids in Malawi and the UK

BMI: body mass index; HDL-C: high density lipoprotein-cholesterol; LDL-C: low density lipoprotein-cholesterol; TC: total cholesterol; TG: triglycerides; WHR: waist-hip ratio
